# New sesquiterpenoids from the South China Sea soft corals *Clavularia viridis* and *Lemnalia flava*

**DOI:** 10.3762/bjoc.15.64

**Published:** 2019-03-15

**Authors:** Qihao Wu, Yuan Gao, Meng-Meng Zhang, Li Sheng, Jia Li, Xu-Wen Li, Hong Wang, Yue-Wei Guo

**Affiliations:** 1 State Key Laboratory of Drug Research, Shanghai Institute of Materia Medica, Chinese Academy of Sciences, 555 Zu Chong Zhi Road, Zhangjiang Hi-Tech Park, Shanghai 201203, P. R. China; 2 College of Pharmaceutical Science and Collaborative Innovation Center of Yangtze River Delta Region Green Pharmaceuticals, Zhejiang University of Technology, Hangzhou 310014, P. R. China

**Keywords:** *Clavularia viridis*, *Lemnalia flava*, NF-κB, PTP1B, sesquiterpenoid, soft coral, terpenes

## Abstract

A detailed chemical investigation of the South China Sea soft corals *Clavularia viridis* and *Lemnalia flava* yielded four new halogenated laurane-type sesquiterpenoids, namely, isobromolaurenisol (**1**), clalaurenol A (**2**), *ent*-laurenisol (**3**), clalaurenol B (**4**), and the new aromadendrane-type sesquiterpenoid claaromadendrene (**6**), together with three known sesquiterpenoids (**5**, **7**, and **8**). Their structures were determined by extensive spectroscopic analysis and by comparison with the previously reported analogues. In a bioassay, compounds **1**, **2** and **4** exhibited interesting inhibitory activities in vitro against PTP1B and NF-κB.

## Introduction

Marine soft corals are important sources of biologically active compounds, which made them attractive targets for natural product chemists. Soft corals of the genus *Clavularia* (class Octocorallia, order Alcyonacea, family Clavulariidea), are prolific sources of numerous biologically active compounds [1–4]. A variety of structurally unique sesquiterpenes, including aromadendranes [5], maalianes [5], elemanes [6], and trinor-guaianes [7–9], have been isolated since the early 1980s from several species of *Clavularia*. Soft corals of the genus *Lemnalia* are also a rich source of sesquiterpenoids and diterpenoids with various intriguing carbon skeletons, such as nardosinanes, neolemnanes, and ylanganes [10]. Many of these secondary metabolites have attracted a lot of attention for further synthetic and pharmacological studies due to their potent bioactivities ranging from neuroprotective, cytotoxic, to anti-inflammatory properties [10].

**Figure 1:**
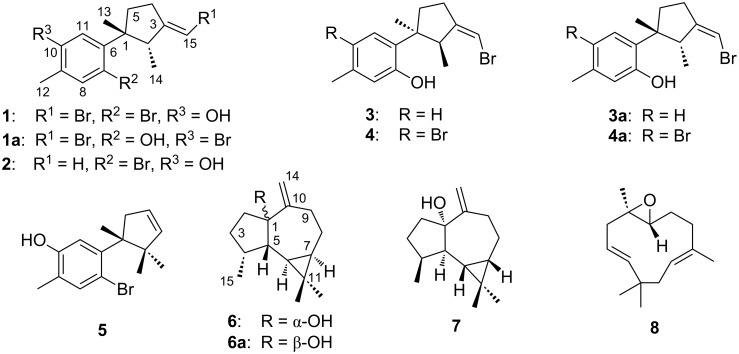
Structures of compounds **1**–**8**.

In the framework of our ongoing research for the bioactive metabolites from South China Sea soft corals [11–12], we made the collection of the title samples *Clavularia viridis* and *Lemnalia flava* off the Xisha Islands, Hainan Province, China. The chemical investigation of two title animals led to the isolation of four new halogenated laurane-type sesquiterpenoids **1**–**4**, one new aromadendrane-type sesquiterpenoid **6**) together with three related known compounds **5**, **7** and **8** (Figure 1). Herein, the isolation, structure elucidation and bioactivity evaluation of these compounds are presented.

## Results and Discussion

The frozen bodies of the two soft corals *C. viridis* and *L. flava* were cut into pieces and exhaustively extracted with acetone. The Et_2_O-soluble portion of the acetone extracts were chromatographed repeatedly over silica gel, Sephadex LH-20, and RP-HPLC to yield pure compounds. A total of eight compounds including compounds **1** (1.0 mg), **2** (0.9 mg), **3** (3.4 mg), **4** (1.4 mg), **5** (0.9 mg), **6** (2.8 mg), **7** (7.8 mg), and **8** (6.8 mg) were obtained from the *C. viridis* sample while two compounds **3** (8.6 mg) and **4** (2.3 mg) were obtained from *L. flava*. Among them, the known compounds were readily identified as cupalaurenol (**5**) [13], 1-hydroxyalloaromadendrene (**7**) [14], and humulene epoxide II (**8**) [15] by comparing their NMR spectroscopic data and optical rotation with those reported in the literature.

**Figure 2:**
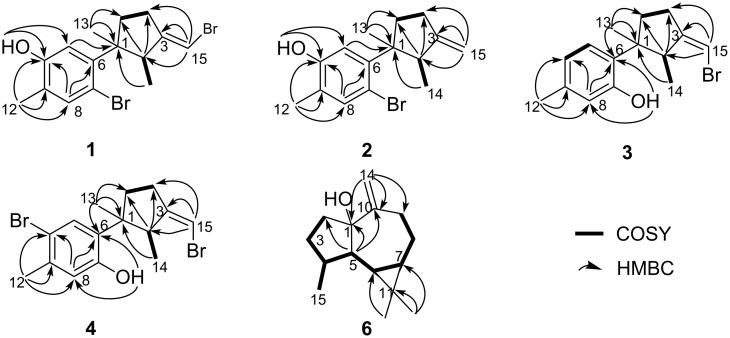
^1^H,^1^H COSY and key HMBC correlations of compounds **1**–**4** and **6**.

Isobromolaurenisol (**1**) was obtained as an optically active colorless oil. Its molecular formula, C_15_H_18_OBr_2_, was deduced by HR-ESIMS with ion peaks at *m*/*z* 370.9657, [M − H]^–^ (calcd for C_15_H_17_OBr_2_, 370.9646), indicating six degrees of unsaturation. The ^13^C NMR and DEPT spectra contained signals attributable to three methyls, two sp^3^ methylenes, one sp^3^ methine, one sp^3^ quaternary carbon, three sp^2^ methines, and five sp^2^ quaternary carbons (Table 1). The typical resonances at δ_C_ 145.6, δ_C_ 113.0, δ_H/C_ 7.30/136.8, δ_C_ 123.4, δ_C_ 153.0, δ_H/C_ 6.71/116.8 revealed the presence of a 1,2,4,5-tetrasubstituted benzene ring, and the signals at δ_H/C_ 6.08/99.1, δ_C_ 154.2 indicated the existence of a trisubstituted double bond. All the above evidence suggested the laurane nature of this molecule, and literature research revealed that **1** should be an isomer of a known laurane-type terpenoid bromolaurenisol (**1a**) [16–17] due to their extremely similar NMR data and the same molecular weight (Figure 1). In fact, the main difference between **1** and **1a** happened only at the tetrasubstituted benzene ring with the substituents exchange between C-7 and C-10 (Figure 1). The assignment of the planar structure of **1** has been further confirmed by 2D NMR experiments, including ^1^H,^1^H COSY, HSQC, and HMBC, with the key correlations shown in Figure 2. In particular, the hydroxy group (δ_H_ 4.68, s) was confirmed to be attached at C-10 by the clear HMBC correlation from O*H* to C-10 and C-11.

**Table 1:** ^1^H and ^13^C NMR data of **1**–**3**^a^ recorded in CDCl_3_.

No.	**1**		**2**		**3**	
	δ_H_ mult (*J* in Hz)	δ_C_	δ_H_ mult (*J* in Hz)	δ_C_	δ_H_ mult (*J* in Hz)	δ_C_

1	–	52.0, qC	–	51.1, qC	–	48.4, qC
2	3.56, q (7.2)	47.5, CH	3.41, q (7.2)	47.1, CH	3.37, q (7.1)	48.1, CH
3	–	154.2, qC	–	157.6, qC	–	153.6, qC
4	2.40, m; 2.51, m	29.0, CH_2_	2.48, m	28.2, CH_2_	2.49, m	28.0, CH_2_
5	1.88, m; 2.34, m	35.1, CH_2_	1.79, m; 2.27, m	36.1, CH_2_	1.93, m; 2.34, m	34.9, CH_2_
6	–	145.6, qC	–	145.6, qC	–	130.4, qC
7	–	113.0, qC	–	113.3, qC	–	153.0, qC
8	7.30, s	136.8, CH	7.31, s	136.7, CH	6.52, s	116.6, CH
9	–	123.4, qC	–	123.1, qC	–	137.2, qC
10	–	153.0, qC	–	152.9, qC	6.71, d (7.7)	121.5, CH
11	6.71, s	116.8, CH	6.73, s	116.8, CH	7.01, d (7.8)	128.2, CH
12	2.18, s	15.0, CH_3_	2.18, s	15.0, CH_3_	2.28, s	20.7, CH_3_
13	1.29, s	25.0, CH_3_	1.30, s	25.1, CH_3_	1.23, s	25.7, CH_3_
14	0.74, d (7.3)	19.2, CH_3_	0.73, d (7.2)	19.9, CH_3_	0.76, d (7.2)	15.0, CH_3_
15	6.08, s	99.1, CH	4.89, s; 4.99, s	106.9, CH_2_	5.93, s	97.5, CH
OH	4.68, s	–	4.67, s	–	4.66, s	–

^a^Bruker DRX-500 spectrometer (125 MHz for ^13^C NMR and 500 MHz for ^1^H NMR) in CDCl_3_, chemical shifts (ppm) referred to CHCl_3_ (δ_C_ 77.16; δ_H_ 7.26); assignments were deduced by analysis of 1D and 2D NMR spectra.

**Figure 3:**
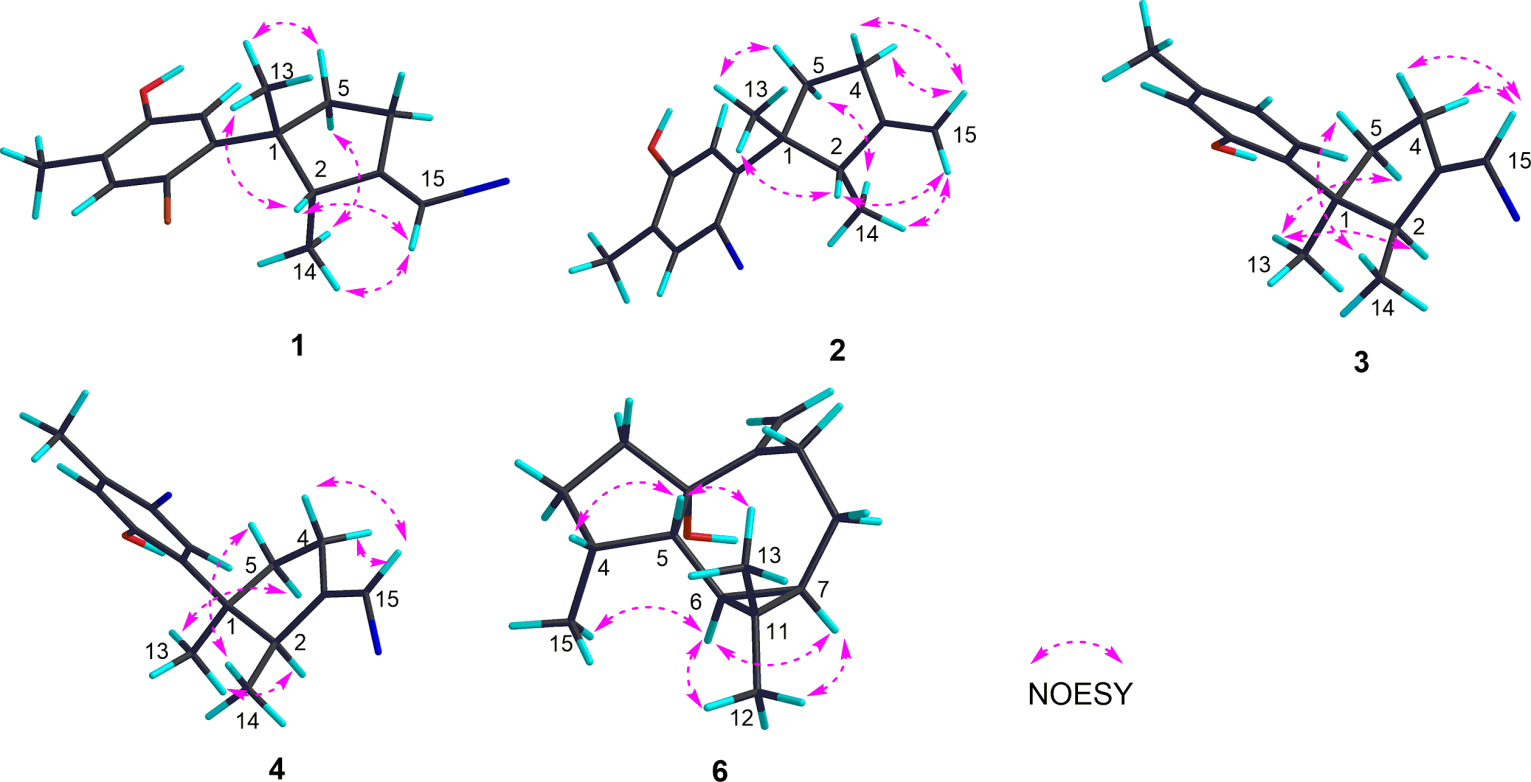
Key NOESY correlations for compounds **1**–**4** and **6**.

The relative configuration of **1** was established by a NOESY experiment (Figure 3), in which the correlations of H_3_-13 (δ_H_ 1.29, s) with H-2 (δ_H_ 3.56, q, *J* = 7.2 Hz) and H-5β (δ_H_ 2.34, m) indicated that these protons were on the same side of the molecule and were tentatively assigned to be β-oriented, while the correlation of H-5α (δ_H_ 1.88, m) and H_3_-14 (δ_H_ 0.74, d, *J* = 7.3) at C-2 indicated CH_3_-14 was α-oriented. Besides, the trisubstituted olefin (Δ^3/15^) was determined to be in *E* configuration due to the clear NOE correlations of H-15 with H_3_-13 and H_3_-14. In view of the above evidences, the relative configuration of compound **1** was determined as 1R*,2R*, the same as **1a** [16–17].

Compound **2** was isolated as an optically active colorless oil. The molecular formula, C_15_H_19_OBr, was established by the molecular ion peak at *m/z* 293.0548, [M − H]^−^ (calcd for C_15_H_18_OBr 293.0541) in the HR-ESIMS spectrum. The ^1^H and ^13^C NMR spectra showed great similarities with those of the co-occurring **1**, which indicated the same laurane skeleton. In fact, compound **2** differed from **1** only by the debromonation at the C-15 position, which was in agree with the lack of 78/80 units in its mass compared to that of **1**. The planar structure of **2** was further confirmed by its 2D NMR data (Figure 2). The relative configurations of the chiral centers on the cyclopentane ring were determined to be the same as **1** by inspection of the proton coupling constants (Table 1) and NOESY experiments (Figure 3). Thus, compound **2** was determined to be the debrominated derivative of **1**, namely, clalaurenol A.

Compound **3** was observed as an optically active colorless oil. The molecular formula, C_15_H_19_OBr, was deduced by HR-EIMS ion peak at *m*/*z* 294.0617, [M]^+^ (calcd for C_15_H_19_OBr, 294.0619). The ^1^H and ^13^C NMR data (Table 1) of **3** were found to be identical to those of laurenisol (**3a**), a halogenated sesquiterpenoid previously isolated from the red alga *Laurencia glandulifera* Kützing [18]. The relative configuration of **3** was established by NOESY correlations (Figure 3) in which the correlations of H_3_-13 (δ_H_ 1.23, s) with H-2 (δ_H_ 3.37, q, *J* = 7.1 Hz) and H-5a (δ_H_ 1.93, m); H_3_-14 (δ_H_ 0.76, d, *J* = 7.2 Hz) with H-5b (δ_H_ 2.34, m) indicating that two methyls at C-1 and C-2 were on the opposite side of the molecule. Besides, the NOESY correlation of one olefin proton (H-15, δ_H_ 5.93, s) and two protons at C-4 (δ_H_ 2.49, m), suggesting the *Z* geometry of the double bond at C-3/C-15. Finally, the sign of the [α]_D_ values of **3** {[α]_D_^20^ −24.2 (*c* 0.06, CHCl_3_); [α]_D_^20^ −16.0 (*c* 0.10, MeOH)} were found to be opposite to that of laurenisol (+85.9) [18]. Thus, compound **3** can be assigned as the enantiomer of **3a**, named *ent*-laurenisol.

Clalaurenol B (**4**) was obtained as an optically active colorless oil. The molecular formula, C_15_H_18_OBr_2_, the same as **4a** [18], was established by HR-ESIMS ion peaks at *m*/*z* 370.9654, [M − H]^−^ (calcd for C_15_H_17_OBr_2_, 370.9646). The ^1^H and ^13^C NMR data of **4** were identical to those of **4a**, a C-10 bromonated analogue of **3a**. In addition, the NOE correlations between H_3_-13 (δ_H_ 1.21, s) with H-2 (δ_H_ 3.34, q, *J* = 7.3 Hz) and H-5a (δ_H_ 1.91, m); H_3_-14 (δ_H_ 0.76, d, *J* = 7.2 Hz) with H-5b (δ_H_ 2.32, m); a proton at C-15 (H-15, δ_H_ 5.94, s) and two protons at C-4 (δ_H_ 2.48, m) suggested the relative configuration (C-1, C-2 and Δ^3,15^) of **4** is the same as **3**. Moreover, the sign of their [α]_D_ values {[α]_D_^20^ −52.1 (*c* 0.08, CHCl_3_); [α]_D_^20^ −22.5 (*c* 0.10, MeOH)} for **4** and {[α]_D_^20^ +74 (*c* 0.58, CHCl_3_)} for **4a**, indicating that compound **4** should be the enantiomer of **4a** [18].

Compound **6** was isolated as an optically active colorless oil. The molecular formula C_15_H_24_O, the same as **7** [14] and **6a** [19–20], was established by HR-ESIMS ion peak at *m*/*z* 220.1824 [M]^+^ (calcd for C_15_H_24_O, 220.1825). A detailed analysis of 2D NMR experiments (Figure 2), revealed that compound **6** had the same planar structure as **6a** and co-occurring **7** differing only in the stereochemistry. The relative configuration of **6** was established by NOESY correlations (Figure 3) in which the correlations of H-6 (δ_H_ 0.62, dd, *J* = 11.4, 9.1 Hz) with H-7 (δ_H_ 0.81, m) and H_3_-15 (δ_H_ 1.01, d, *J* = 7.2 Hz); H_3_-12 (δ_H_ 1.08, s) with H-6 and H-7, indicating that these protons were on the same side of the molecule and were tentatively assigned to be α-oriented, while correlations of H-5 (δ_H_ 1.60, m) with H-4 (δ_H_ 2.20, m) and H_3_-13 (δ_H_ 0.99, s) suggesting these protons were on the opposite orientation. In view of the above evidences, the relative configuration of compound **6** was determined as 4R*,5S*,6R*,7R*. In fact, the only difference between compounds **6** and **6a** was the configuration of the hydroxy group at C-1 with α-orientation for **6** while β-orientation for **6a** [19–20]. Further, due to the influence of the configuration inversions of C-1, the ^13^C NMR chemical shift of the carbon at C-1 (δ_C_ 85.5, qC), was apparently upfield shifted (Δδ = −3.0) comparing to compound **6a** (Table 2), giving the further support of the assigned structure for **6** (Figure 1). Thus, compound **6** was determined as a C-1 isomer of *ent*-1-hydroxyalloaromadendrene (**6a**), namely, claaromadendrene.

**Table 2:** ^1^H and ^13^C NMR data of **4**^a^ and **6**^a^ and ^13^C NMR data of **6a**^b^ and **7**^a^ recorded in CDCl_3_.

No.	**4**		**6**		**6a**^b^	**7**
	δ_H_ mult (*J* in Hz)	δ_C_	δ_H_ mult (*J* in Hz)	δ_C_	δ_H_ mult (*J* in Hz)	δ_C_

1	–	48.4, qC	–	85.5, qC	88.5, qC	88.7, qC
2	3.34, q (7.3)	47.9, CH	1.76, m; 1.96, m	37.1, CH_2_	36.5, CH_2_	36.6, CH_2_
3	–	153.1, qC	1.55, m; 1.90, m	33.3, CH_2_	30.6, CH_2_	30.7, CH_2_
4	2,48, m	27.9, CH_2_	2.20, m	34.9, CH	34.3, CH	34.4, CH
5	1.91, m; 2.32, m	34.9, CH_2_	1.60, m	46.8, CH	49.0, CH	49.1, CH
6	–	133.2, qC	0.62, dd (11.4, 9.1)	21.8, CH	23.3, CH	23.4, CH
7	–	152.3, qC	0.81, m	27.5, CH	25.4, CH	25.5, CH
8	6.59, s	118.1, CH	0.98, m; 2.02, m	25.2, CH_2_	21.2, CH_2_	21.4, CH_2_
9	–	136.5, qC	2.27, m; 2.47, dd (13.9, 12.6)	34.1, CH_2_	32.0, CH_2_	32.2, CH_2_
10	–	115.5, qC	–	155.1, qC	152.9, qC	153.0, qC
11	7.23, s	131.9, CH	–	19.8, qC	not detected	18.1, qC
12	2.30, s	22.3, CH_3_	1.08, s	29.0, CH_3_	28.6, CH_3_	28.7, CH_3_
13	1.21, s	25.4, CH_3_	0.99, s	15.7, CH_3_	15.9, CH_3_	16.0, CH_3_
14	0.76, d (7.2)	15.0, CH_3_	4.66, t (1.6); 4.80, d (1.7)	108.3, CH_2_	111.7, CH_2_	111.8, CH_2_
15	5.94, s	97.8, CH	1.01 d, (7.2)	18.8, CH_3_	16.6, CH_3_	16.7, CH_3_
OH	4.75, s	–	–	–		

^a^Bruker DRX-500 spectrometer (125 MHz for ^13^C NMR and 500 MHz for ^1^H NMR) in CDCl_3_, chemical shifts (ppm) referred to CHCl_3_ (δ_C_ 77.16; δ_H_ 7.26); assignments were deduced by analysis of 1D and 2D NMR spectra. ^b^Data reported in ref. [19] (in CDCl_3_).

In bioassays, all the isolated compounds were tested for protein tyrosine phosphase-1B (PTP1B) and NF-κB inhibitory activity. In the PTP1B inhibitory assay, the inhibitory effects of compounds **1**–**8** were evaluated against PTP1B, and the result showed that compounds **1**, **2** and **4** had a moderate PTP1B inhibitory activity with IC_50_ values of 18.8, 21.8 and 15.6 μM, respectively. The known PTP1B inhibitor oleanolic acid (IC_50_ = 3.0 μM) were used as positive control in this assay. In NF-κB inhibitory assay, compounds **2** and **4** showed the most potent NF-κB signaling pathway inhibition with IC_50_ values of 6.8 and 7.3 μM, respectively, while compound **1** showed moderate activity with an IC_50_ value of 19.9 μM (Table 3).

**Table 3:** PTP1B and NF-κB inhibitory effect of compounds **1**–**8**.

Compounds	IC_50_ (μM)	
	PTP1B	NF-κB

**1**	18.8	19.9
**2**	21.8	6.8
**3**	–	–
**4**	15.6	7.3
**5**	–	–
**6**	–	–
**7**	–	–
**8**	–	–
**A**^#^	3.0	–
**B**^#^	–	14.0

**A^#^** and **B^#^**, representing oleanolic acid and bortezomib, respectively, were used as the positive controls.

## Conclusion

In summary, eight sesquiterpenoids (**1**–**8**), belonging to four different structural types, were isolated from two South China Sea soft corals (*C. viridis* and *L. flava*) for the first time. The discovery of these metabolites extended the structural diversity and complexity of sesquiterpenoids derived from soft corals *C. viridis* and *L. flava*. In fact, to our knowledge, naturally occurring laurane- (**1**–**4**) and cuparane-derived (**5**) sesquiterpenoids, are extremely rare in soft corals. Previously, such sesquiterpenoids have only been isolated from the red algae of the genus *Laurencia* [14,16–17,21] and some sea hares that prey on it [13,22]. In this paper, the chemical investigation of two different soft corals collected off the South China Sea, which belong to two different genera, have resulted in the discovery of two common new halogenated laurane-type sesquiterpenoids (**3** and **4**). Based on these findings, other than prey–predator relationship, the common symbiotic organisms in the algae and the soft corals might be the source of these metabolites. In fact, many investigations have proved that [23] numerous natural products are actually produced by microbes and/or microbial interactions with the “host from whence it was isolated”. Further chemical investigation of these soft corals in the South China Sea as well as their associated microorganisms should be conducted to verify the true origin of these metabolites and to further understand the real biological/ecological roles they played in the life cycle of the title animals in the South China Sea.

The promising PTP1B inhibitory activity of laurane-type sesquiterpenoids [24] in a previously report from our group, inspired us to test the PTP1B inhibitory activity of compounds **1**–**4**. Among them, compound **3** was inactive against PTP1B enzyme, whereas compounds **1**, **2** and **4** exhibited considerable PTP1B inhibitory activity with IC_50_ values of 18.8, 21.8, and 15.6 μM, respectively. Compounds **1**, **2** and **4** also showed strong NF-κB inhibitory activity with IC_50_ values of 19.9, 6.8 and 7.3 μM, respectively. With regard to their structure–activity relationship, the bromine atom on the benzene ring may play the key functional role in the inhibitory activity. This study could thus provide a clue for the further biological study and structure modification of marine brominated laurane sesquiterpenoid derivatives towards new effective PTP1B and/or NF-κB inhibitors.

## Experimental

### General experimental procedures

Optical rotations were measured on a Perkin-Elmer 241MC polarimeter. IR spectra were recorded on a Nicolet-Magna FT-IR 750 spectrometer. EIMS and HR-EIMS spectra were recorded on a Finnigan-MAT-95 mass spectrometer. HR-ESIMS spectra were recorded on a Q-TOF Micro LC–MS–MS mass spectrometer. The NMR spectra were measured on a Bruker DRX-500 spectrometer with the residual CHCl_3_ (δ_H_ 7.26 ppm, δ_C_ 77.2 ppm) as internal standard. Chemical shifts are expressed in δ (ppm) and coupling constants (*J*) in Hz. ^1^H and ^13^C NMR assignments were supported by ^1^H,^1^H COSY, HSQC, HMBC and NOESY experiments. Commercial silica gel (Qing Dao Hai Yang Chemical Group Co., 300–400 and 500–600 mesh) and Sephadex LH-20 (Amersham Biosciences) were used for column chromatography. Precoated silica gel GF254 plates (Sinopharm Chemical Reagent Co., Shanghai, China) were used for TLC. Reversed-phase (RP) HPLC purification was carried out on an Agilent 1260 series liquid chromatography system equipped with a DAD G1315D detector at 210 and 254 nm and with a semi-preparative ODS-HG-5 column [5 μm, 250 × 9.4 mm]. All solvents used for CC were of analytical grade, and solvents used for HPLC were of HPLC grade.

### Collection of biological materials

The soft corals *C. viridis* and *L. flava* were collected by scuba from Xisha Island, Hainan Province, China, in March 23, 2013, at a depth of −15 to −20 m, and identified by Professor Xiu-Bao Li from Hainan University. The voucher samples, both *C. viridis* and *L. flava* are deposited at the Shanghai Institute of Materia Medica, CAS, under registration Nos. 13XS-49 and 13XS-52, respectively.

### Extraction and isolation

The lyophilized bodies of *C. viridis* (80 g, dry weight) were minced into pieces and exhaustively extracted with acetone at room temperature (4 × 1 L). The solvent-free actone extract was partitioned between Et_2_O and H_2_O. The organic phase was evaporated under reduced pressure to give a dark-red residue (1.1 g), which was subjected to a gradient silica gel column chromatography (CC) [Et_2_O/petroleum ether (PE), 0–100%] to yield 6 fractions (A–F). Fraction C was subjected to Sephadex LH-20 CC (PE/CH_2_Cl_2_/MeOH, 2:1:1) to give 4 sub-fractions (C1–C4). Fraction C4 was purified by silica gel CC (500–600 mesh, Et_2_O/PE, 4:96) to afford pure **3** (3.4 mg), **4** (1.4 mg) and **8** (6.8 mg). Fraction D eluted with Sephadex LH-20 CC (PE/CH_2_Cl_2_/MeOH, 2:1:1), followed by CC on silica gel (500–600 mesh, Et_2_O/PE, 5:95) to afford pure **1** (1.0 mg), **2** (0.9 mg) and **5** (0.9 mg). Fraction E gave compounds **6** (2.8 mg) and **7** (7.8 mg) after CC on Sephadex LH-20 (PE/CH_2_Cl_2_/MeOH, 2:1:1) and silica gel (500–600 mesh, Et_2_O/PE, 8:92).

The frozen animals *L. flava* (350 g, dry weight) were cut into pieces and extracted exhaustively with acetone at room temperature (6 × 2.0 L). The organic extract was evaporated to give a brown residue, which was then partitioned between H_2_O and Et_2_O. The upper layer was concentrated under reduced pressure to give a brown residue 8.0 g. The resulted residue was separated into seven fractions (A–G) by gradient silica-gel CC. The resulting fractions were then fractionated into sub-fractions by Sephadex LH-20. The sub-fraction C5 was purified by Semi-preparative HPLC (87% MeOH), yielding compounds **3** (8.6 mg) and **4** (2.3 mg).

Isobromolaurenisol (**1**): Colorless oil; [α]_D_^20^ +33.1 (*c* 0.07, CHCl_3_); ^1^H and ^13^C NMR data, see Table 1; HR-ESIMS *m*/*z*: [M − H]^−^ 370.9657, 372.9635, 374.9619 (calcd for C_15_H_17_OBr_2_, 370.9646).

Clalaurenol A (**2**): Colorless oil; [α]_D_^20^ +69.7 (*c* 0.05, CHCl_3_); ^1^H and ^13^C NMR data, see Table 1; HR-ESIMS *m/z*: [M – H]^−^ 293.0548, 295.0536 (calcd for C_15_H_18_OBr, 293.0541).

*ent*-Laurenisol (**3**): Colorless oil; [α]_D_^20^ −24.2 (*c* 0.06, CHCl_3_); [α]_D_^20^ –16.0 (*c* 0.10, MeOH); ^1^H and ^13^C NMR data, see Table 1; HR-EIMS *m*/*z*: [M]^+^ 294.0617, 296.0594 (calcd for C_15_H_19_OBr, 294.0619).

Clalaurenol B (**4**): Colorless oil; [α]_D_^20^ −52.1 (*c* 0.08, CHCl_3_); [α]_D_^20^ −22.5 (*c* 0.10, MeOH); ^1^H and ^13^C NMR data, see Table 1; HR-ESIMS *m*/*z*: [M − H]^−^ 370.9654, 372.9637, 374.9618 (calcd for C_15_H_17_OBr_2_, 370.9646).

Claaromadendrene **6**: Colorless oil; [α]_D_^20^ −83.0 (*c* 0.20, CHCl_3_); ^1^H and ^13^C NMR data, see Table 1; HR-EIMS *m*/*z*: [M]^+^ 220.1824 (calcd for C_15_H_24_O, 220.1825).

### PTP1B inhibitory activity assay

The recombinant PTP1B catalytic domain was expressed and purified according to a previous report [24]. The enzymatic activities of the PTP1B catalytic domain were determined at 30 °C by monitoring the hydrolysis of *p*NPP. Dephosphorylation of *p*NPP generated the product *p*NP, which was monitored at an absorbance of 405 nm with an EnVision multilabel plate reader (Perkin–Elmer Life Sciences, Boston, MA). In a typical 100 L assay mixture containing 50 mmol/L 3-morpholinopropanesulfonic acid, pH 6.5, 2 mmol/L *p*NPP, and 30 nmol/L recombinant PTP1B, activities were continuously monitored and the initial rate of hydrolysis was determined by using the early linear region of the enzymatic reaction kinetic curve. The IC_50_ was calculated with Prism 4 software (Graphpad, San Diego, CA) from the nonlinear curve fitting of the percentage of inhibition (% inhibition) vs the inhibitor concentration [*I*] by using the following equation: % inhibition = 1/(1 + [IC_50_/[*I*]]^k^), where *k* is the Hill coefficient; IC_50_ ≥ 50 μM was considered inactive.

### NF-κB signaling pathway inhibitory activity assays

NF-κB signaling pathway inhibitory activity was evaluated according to the previously reported protocol [25]. Stable HEK293/NF-κB cells were plated into 384-well plates at a concentration of approximate 2500 cells per well. After culturing overnight, compounds were added to the medium at a final concentration of 0.1 μg/mL. HEK293/NF-κB cells were seeded into 96-well cell culture plates (Corning, NY, USA) and allowed to grow for 24 h. The cells were then treated with compounds, followed by stimulation with TNF-α. 6 h later, the luciferase substrate was added to each well, and the released luciferin signal was detected using an EnVision microplate reader. The IC_50_ was calculated with Prism 4 software (Graphpad, San Diego, CA) from the nonlinear curve fitting of the percentage of inhibition (% inhibition) versus the inhibitor concentration [*I*] by using the following equation: % inhibition = 100/(1 + [IC_50_/[*I*]]^k^), where *k* is the Hill coefficient. Bortezomib was used as a positive control with an IC_50_ value of 14.0 μM.

## Supporting Information

File 1 Spectral data of compounds **1–4** and **7**.
