# Supplementary material for: New sesquiterpenoids from the South China Sea soft corals Clavularia viridis and Lemnalia flava
Source: Beilstein J Org Chem. 2019 Mar 15;15:695–702. doi: 10.3762/bjoc.15.64 (PMC6423584; doi:10.3762/bjoc.15.64)
Supplement: File 1 — Spectral data of compounds 1–4 and 7. [file Beilstein_J_Org_Chem-15-695-s001.pdf]

BEILSTEIN JOURNAL OF ORGANIC CHEMISTRY

Supporting Information

for

New sesquiterpenoids from the South China Sea soft corals *Clavularia viridis* and *Lemnalia flava*

Qihao Wu, Yuan Gao, Meng-Meng Zhang, Li Sheng, Jia Li, Xu-Wen Li, Hong Wang and Yue-Wei Guo

Beilstein J. Org. Chem. **2019,** *15,* 695–702. doi:10.3762/bjoc.15.64

Spectral data of compounds 1–4 and 7

License and Terms: This is a supporting information file under the terms of the Creative Commons Attribution License (http://creativecommons.org/licenses/by/4.0). Please note that the reuse, redistribution and reproduction in particular requires that the authors and source are credited.
The license is subject to the *Beilstein Journal of Organic Chemistry* terms and conditions: (https://www.beilstein-journals.org/bjoc)

Contents

Figure S1: ^{1}H NMR spectrum (500 MHz, $CDCl_3$) of **1**

Figure S2: ^{13}C NMR spectrum (125 MHz, $CDCl_3$) of **1**

Figure S3: $^{1}H,^{1}H$ COSY spectrum (500 MHz, $CDCl_3$) of **1**

Figure S4: HMQC spectrum (500 MHz, $CDCl_3$) of **1**

Figure S5: HMBC spectrum (500 MHz, $CDCl_3$) of **1**

Figure S6: NOESY spectrum (500 MHz, $CDCl_3$) of **1**

Figure S7: IR spectrum of **1**

Figure S8: HR-ESIMS spectrum of **1**

Figure S9: ^{1}H NMR spectrum (500 MHz, $CDCl_3$) of **2**

Figure S10: ^{13}C NMR spectrum (125 MHz, $CDCl_3$) of **2**

Figure S11: $^{1}H,^{1}H$ COSY spectrum (500 MHz, $CDCl_3$) of **2**

Figure S12: HMQC spectrum (500 MHz, $CDCl_3$) of **2**

Figure S13: HMBC spectrum (500 MHz, $CDCl_3$) of **2**

Figure S14: NOESY spectrum (500 MHz, $CDCl_3$) of **2**

Figure S15: IR spectrum of **2**

Figure S16: HRESIMS spectrum of **2**

Figure S17: ^{1}H NMR spectrum (500 MHz, $CDCl_3$) of **3**

Figure S18: ^{13}C NMR spectrum (125 MHz, $CDCl_3$) of **3**

Figure S19: $^{1}H,^{1}H$ COSY spectrum (500 MHz, $CDCl_3$) of **3**

Figure S20: HMQC spectrum (500 MHz, $CDCl_3$) of **3**

Figure S21: HMBC spectrum (500 MHz, $CDCl_3$) of **3**

Figure S22: NOESY spectrum (500 MHz, $CDCl_3$) of **3**

Figure S23: IR spectrum of **3**

Figure S24: HR-EIMS spectrum of **3**

Figure S25: ^{1}H NMR spectrum (500 MHz, $CDCl_3$) of **4**

Figure S26: ^{13}C NMR spectrum (125 MHz, $CDCl_3$) of **4**

Figure S27: $^{1}H,^{1}H$ COSY spectrum (500 MHz, $CDCl_3$) of **4**

Figure S28: HMQC spectrum (500 MHz, $CDCl_3$) of **4**

Figure S29: HMBC spectrum (500 MHz, $CDCl_3$) of **4**

Figure S30: NOESY spectrum (500 MHz, $CDCl_3$) of **4**

Figure S31: IR spectrum of **4**

Figure S32: HR-ESIMS spectrum of **4**

Figure S33: ^{1}H NMR spectrum (500 MHz, $CDCl_3$) of **6**

Figure S34: ^{13}C NMR spectrum (125 MHz, $CDCl_3$) of **6**

Figure S35: $^{1}H,^{1}H$ COSY spectrum (500 MHz, $CDCl_3$) of **6**

Figure S36: HMQC spectrum (500 MHz, $CDCl_3$) of **6**

Figure S37: HMBC spectrum (500 MHz, $CDCl_3$) of **6**

Figure S38: NOESY spectrum (500 MHz, $CDCl_3$) of **6**

Figure S39: IR spectrum of **6**

Figure S40: HR-EIMS spectrum of **6**

Figure S1: ^{1}H NMR spectrum (500 MHz, $CDCl_3$) of **1**.

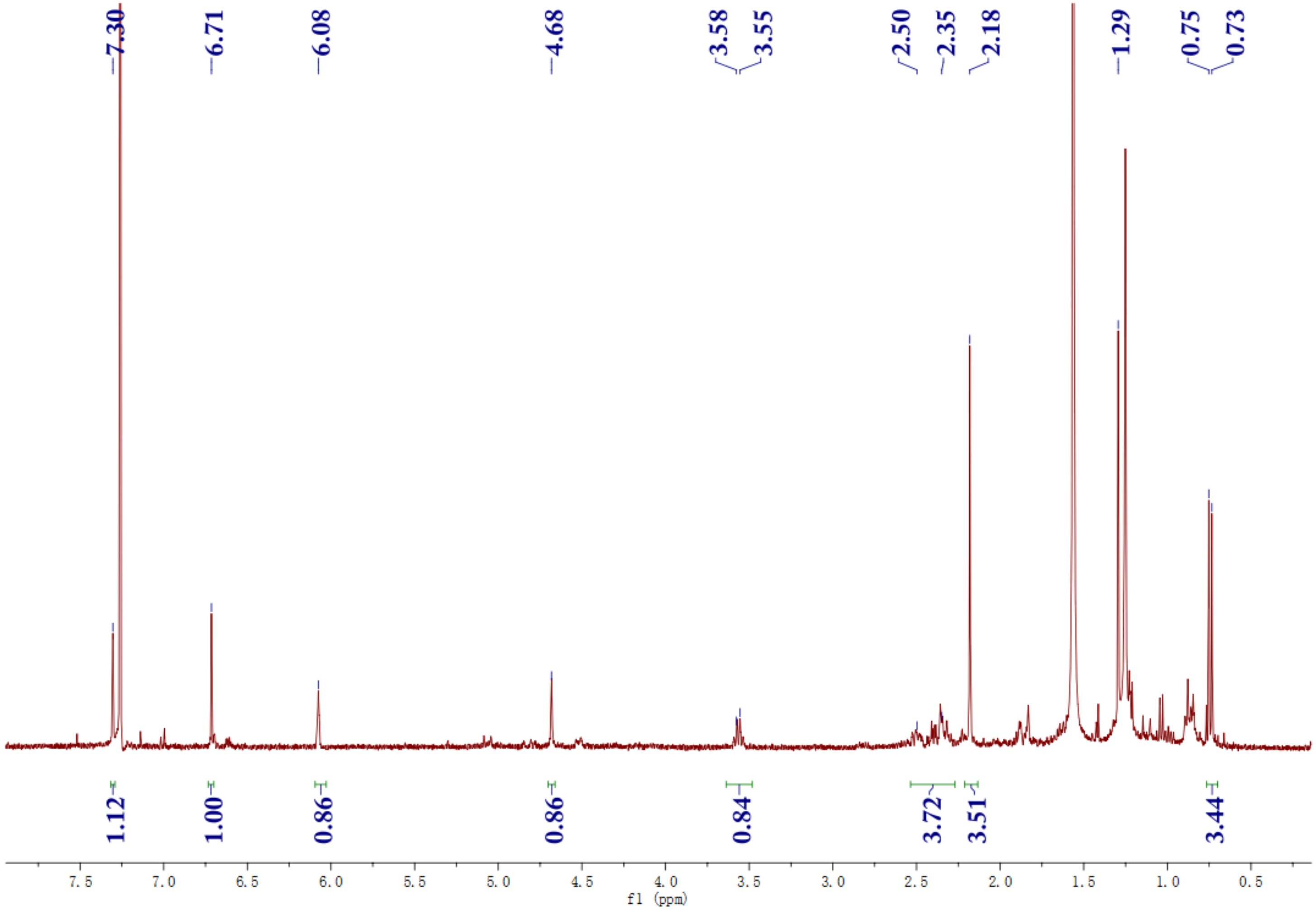

Figure S2: ^{13}C NMR spectrum (125 MHz, $CDCl_3$) of **1**.

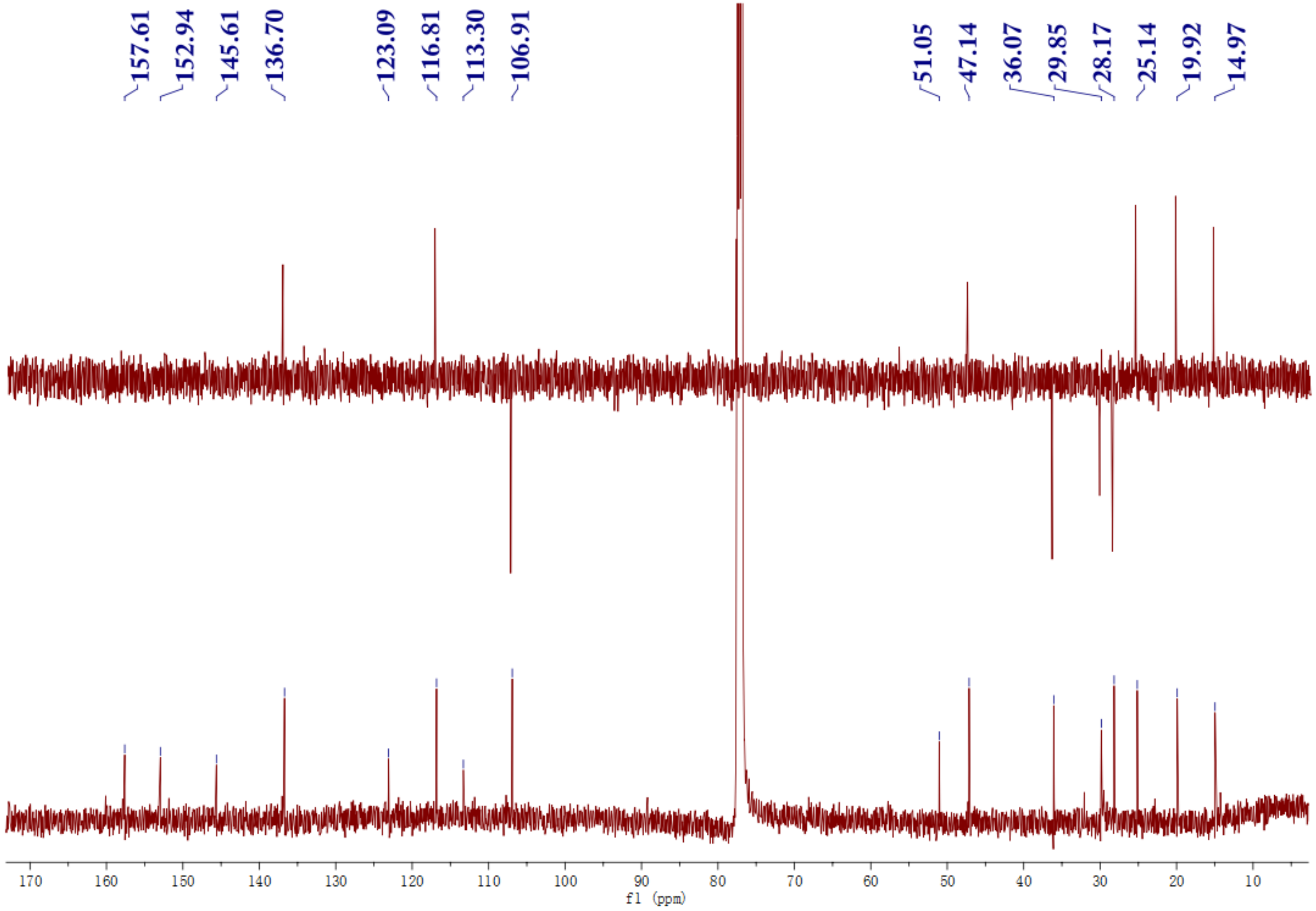

Figure S3: $^{1}H,^{1}H$ COSY spectrum (500 MHz, $CDCl_3$) of **1**.

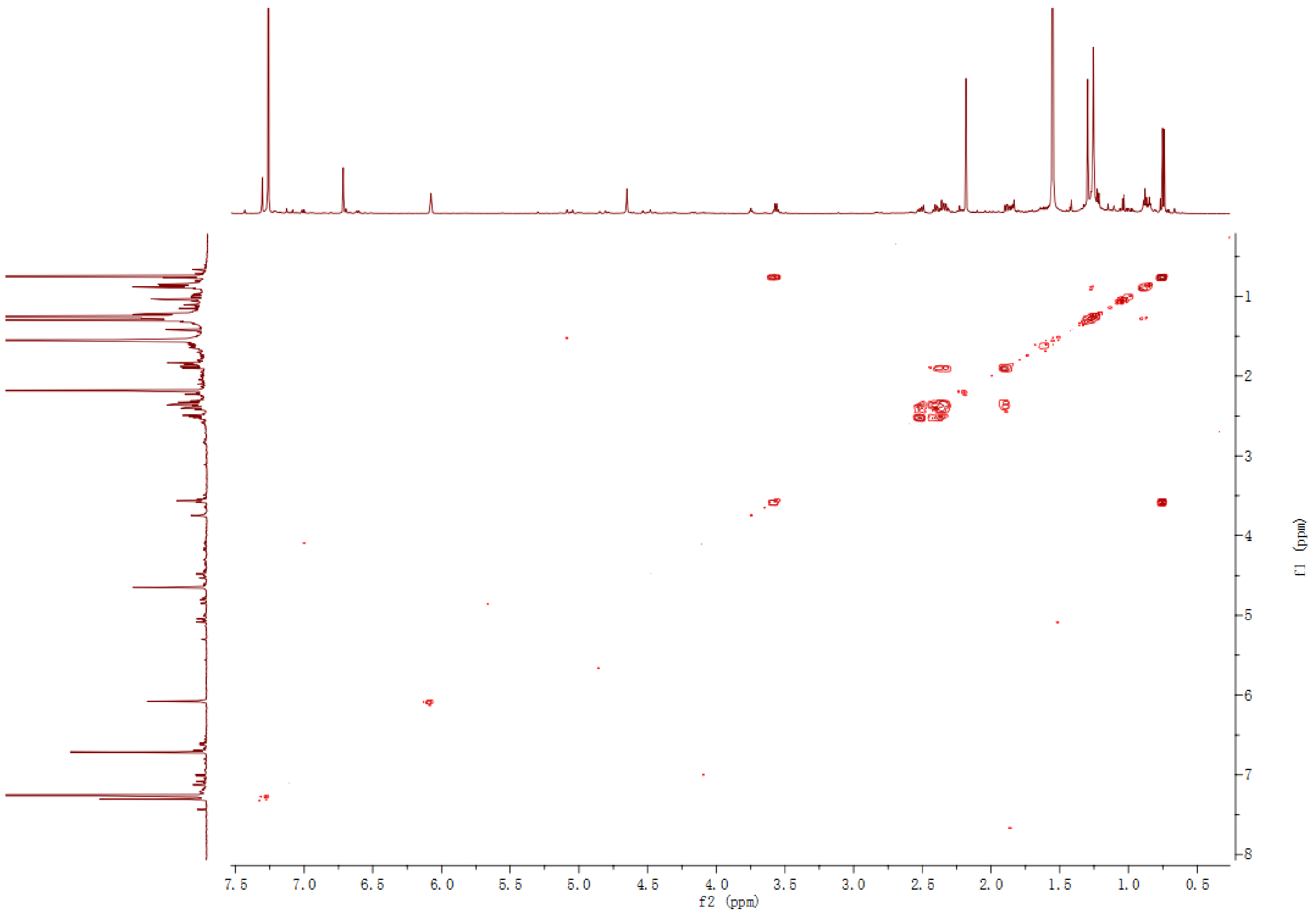

Figure S4: HMQC spectrum (500 MHz, $CDCl_3$) of **1**.

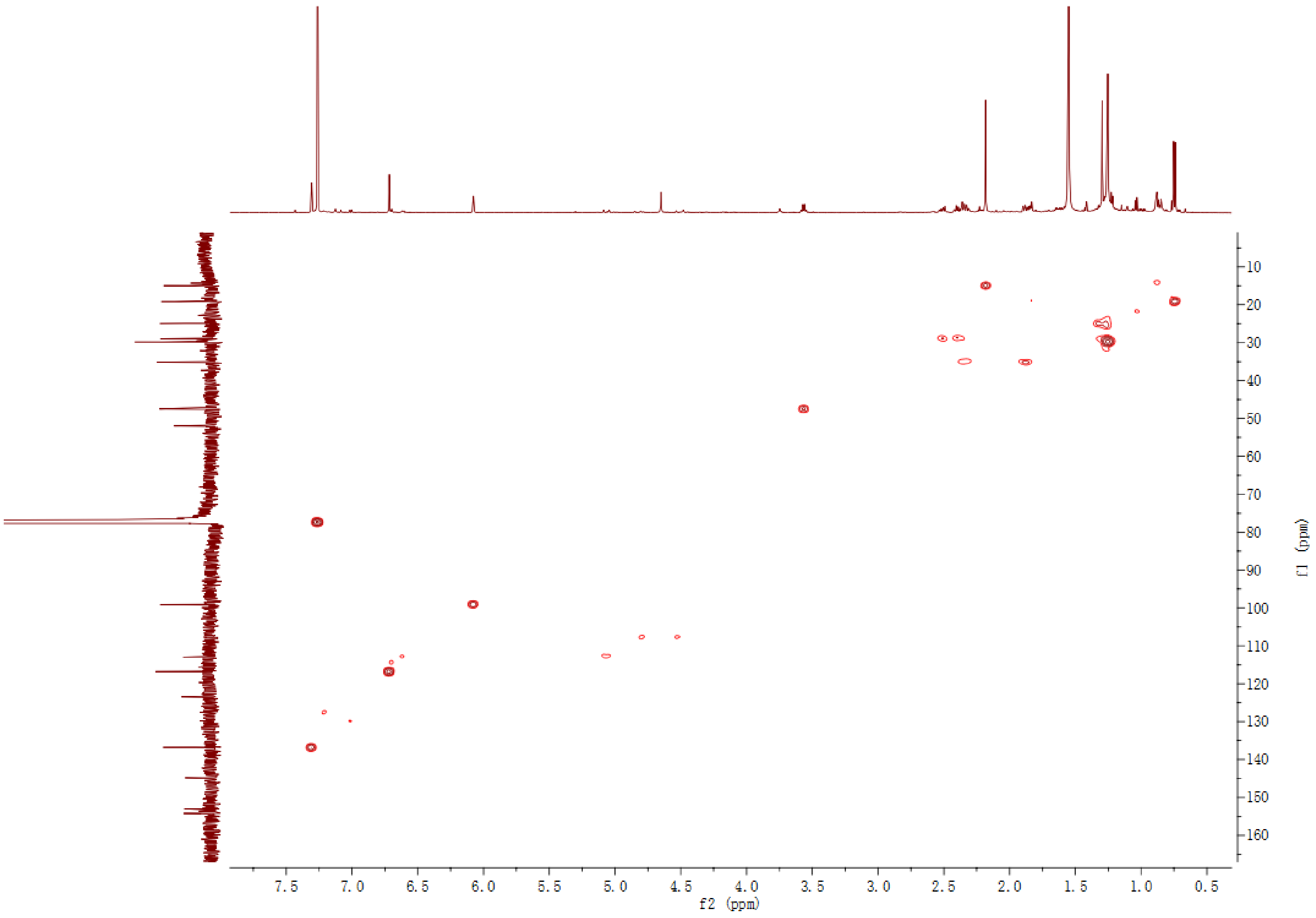

Figure S5: HMBC spectrum (500 MHz, $CDCl_3$) of **1**.

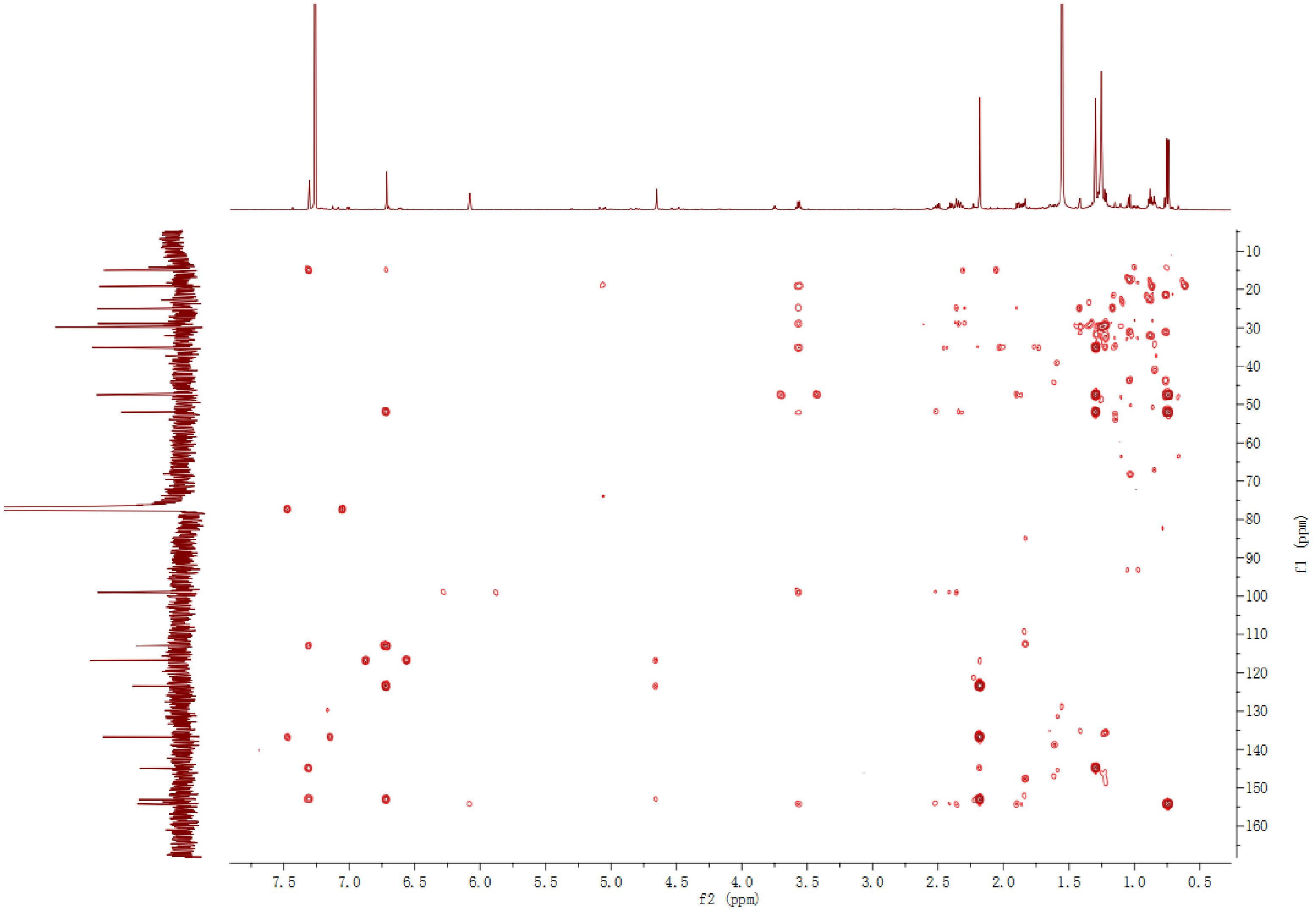

Figure S6: NOESY spectrum (500 MHz, $CDCl_3$) of **1**.

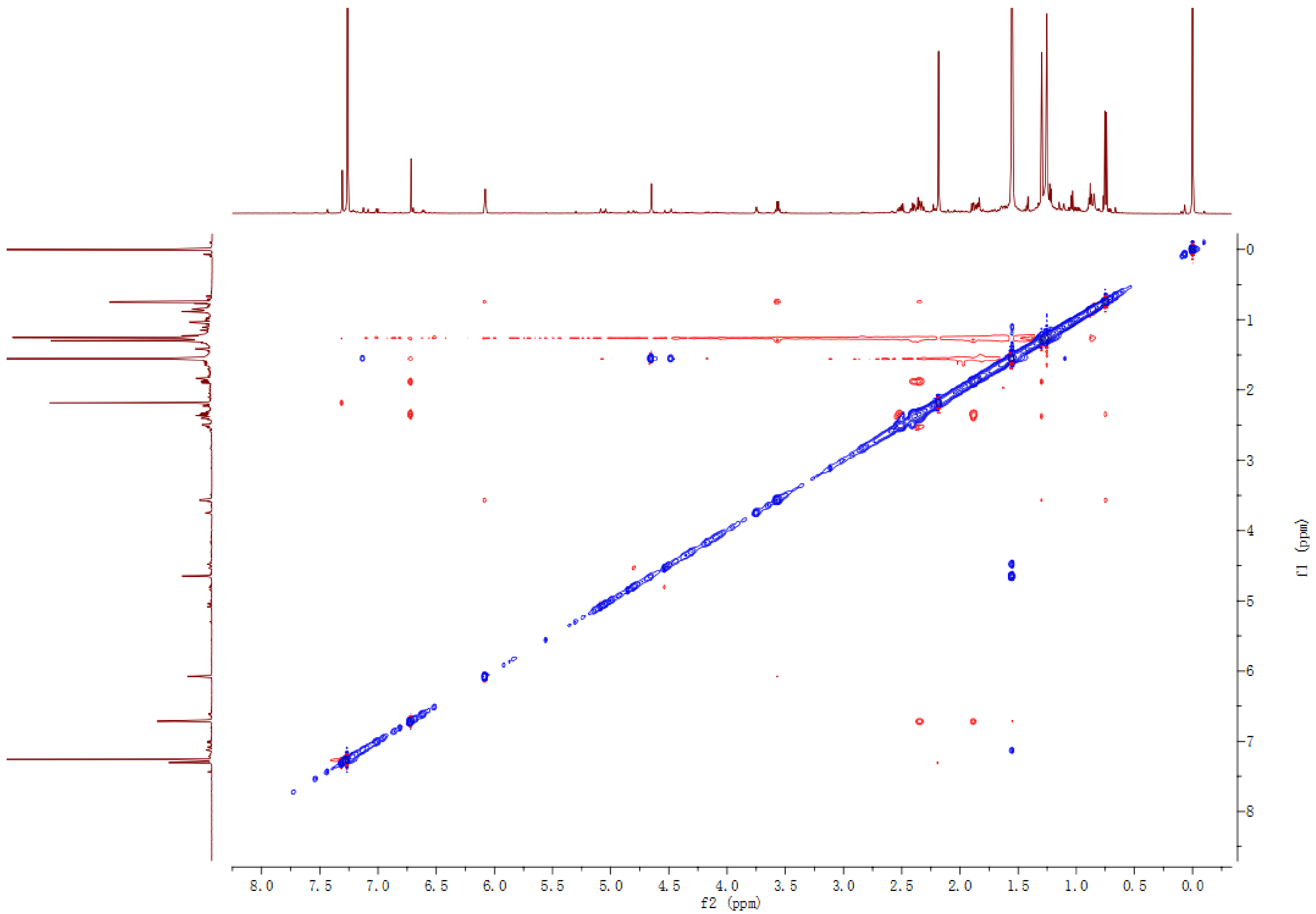

Figure S7: IR spectrum of **1**.

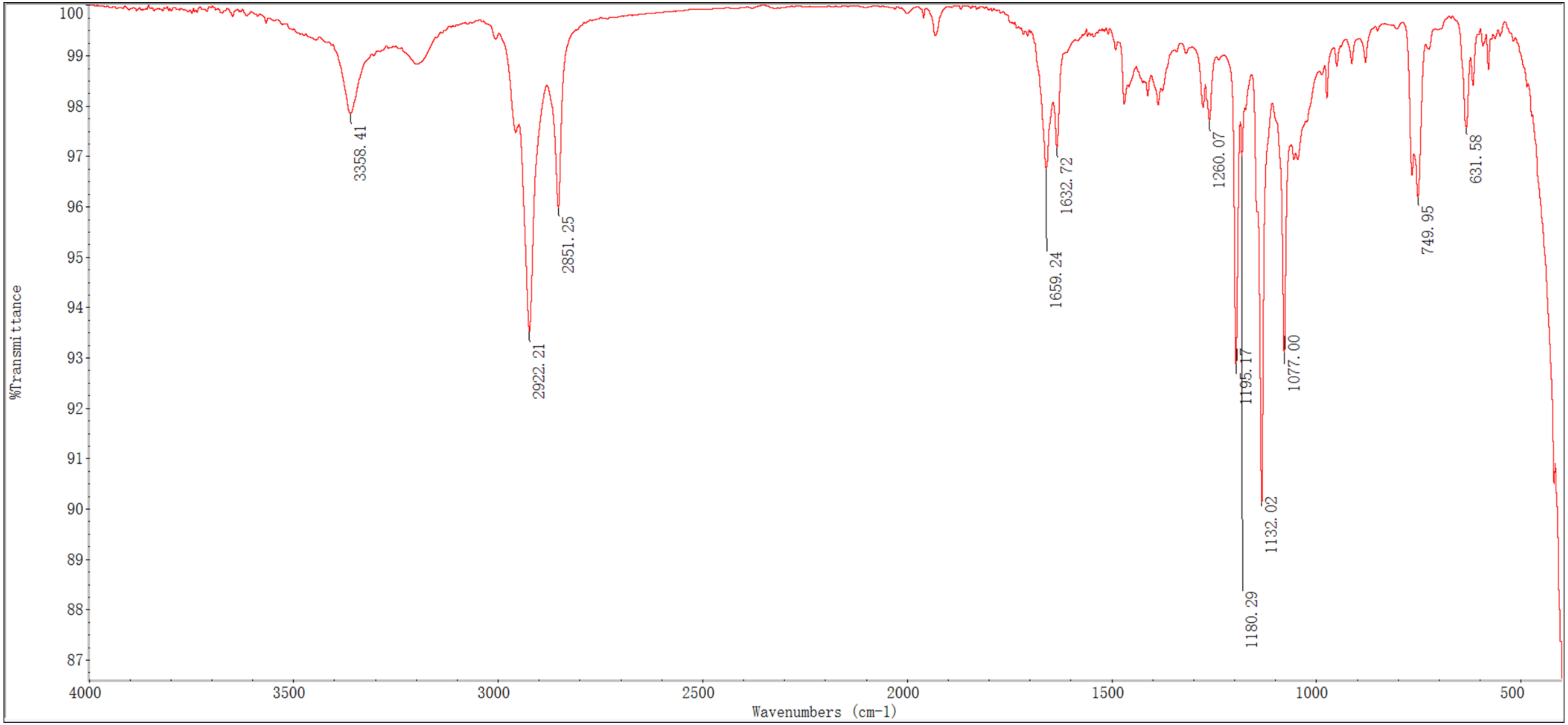

Figure S8: HR-ESIMS spectrum of **1**.

Elemental Composition Report

Page 1

Multiple Mass Analysis: 3 mass(es) processed

Tolerance = 20.0 PPM / DBE: min = -1.5, max = 50.0

Isotope cluster parameters: Separation = 1.0 Abundance = 1.0%

Monoisotopic Mass, Odd and Even Electron Ions

19 formula(e) evaluated with 1 results within limits (up to 20 closest results for each mass)

SIMM-Mass Spec Q-Tof Ultima 30-Sep-2015

D4h2

151297-1 25 (0.479) AM (Cen,4, 80.00, Ht,9000.0,384.93,0.70); Sm (SG, 2x1.00); Cm (25:46) TOF MS ES- 741

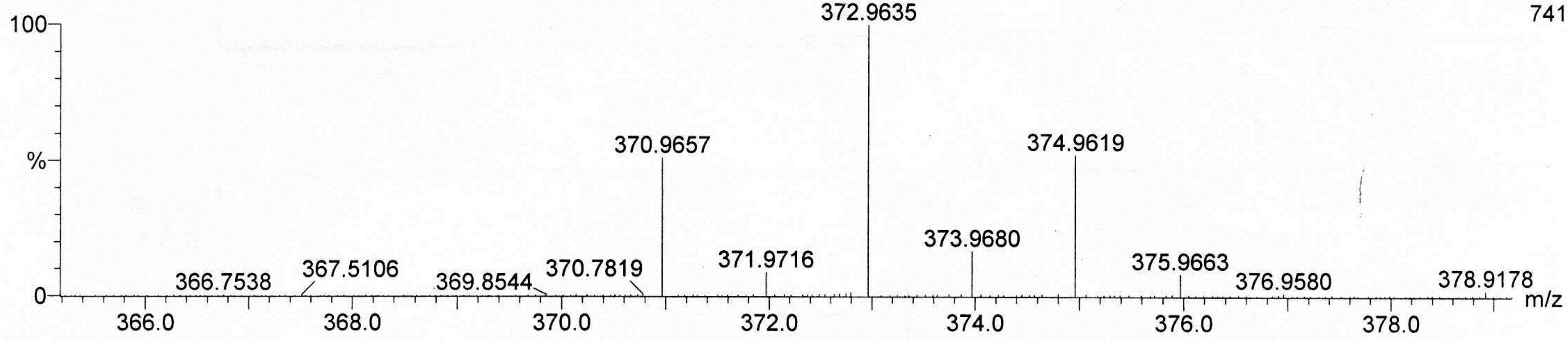

Mass	RA	Calc. Mass	mDa	PPM	DBE	Score	Formula
Minimum:	50.00				-1.5		
Maximum:	100.00		200.0	20.0	50.0		
370.9657	51.04	370.9646	1.1	2.9	6.5	1	C15 H17 O Br2
372.9635	100.00	---					
374.9619	52.28	---					

Figure S9: ^{1}H NMR spectrum (500 MHz, $CDCl_3$) of **2**.

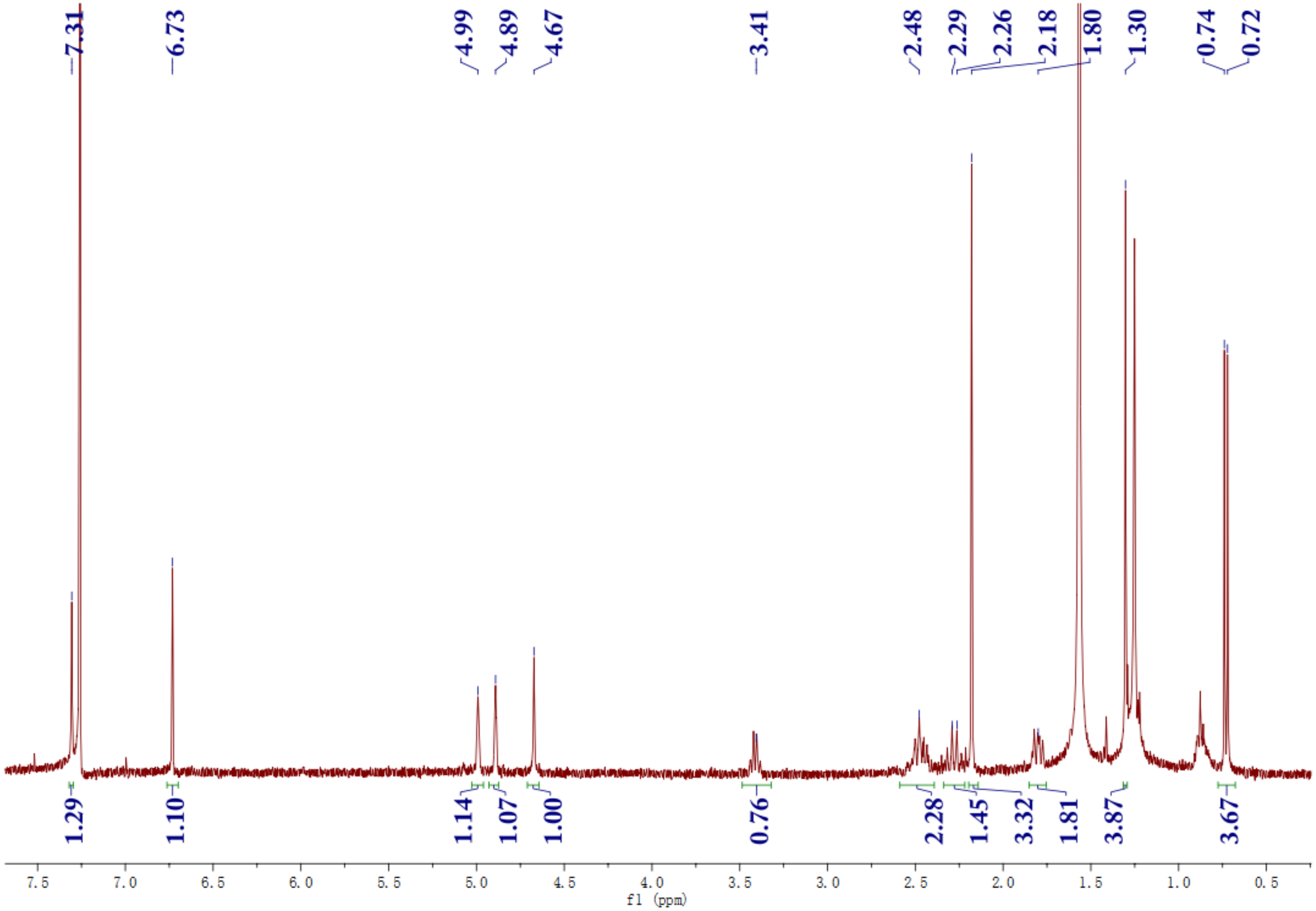

Figure S10: ^{13}C NMR spectrum (125 MHz, $CDCl_3$) of **2**.

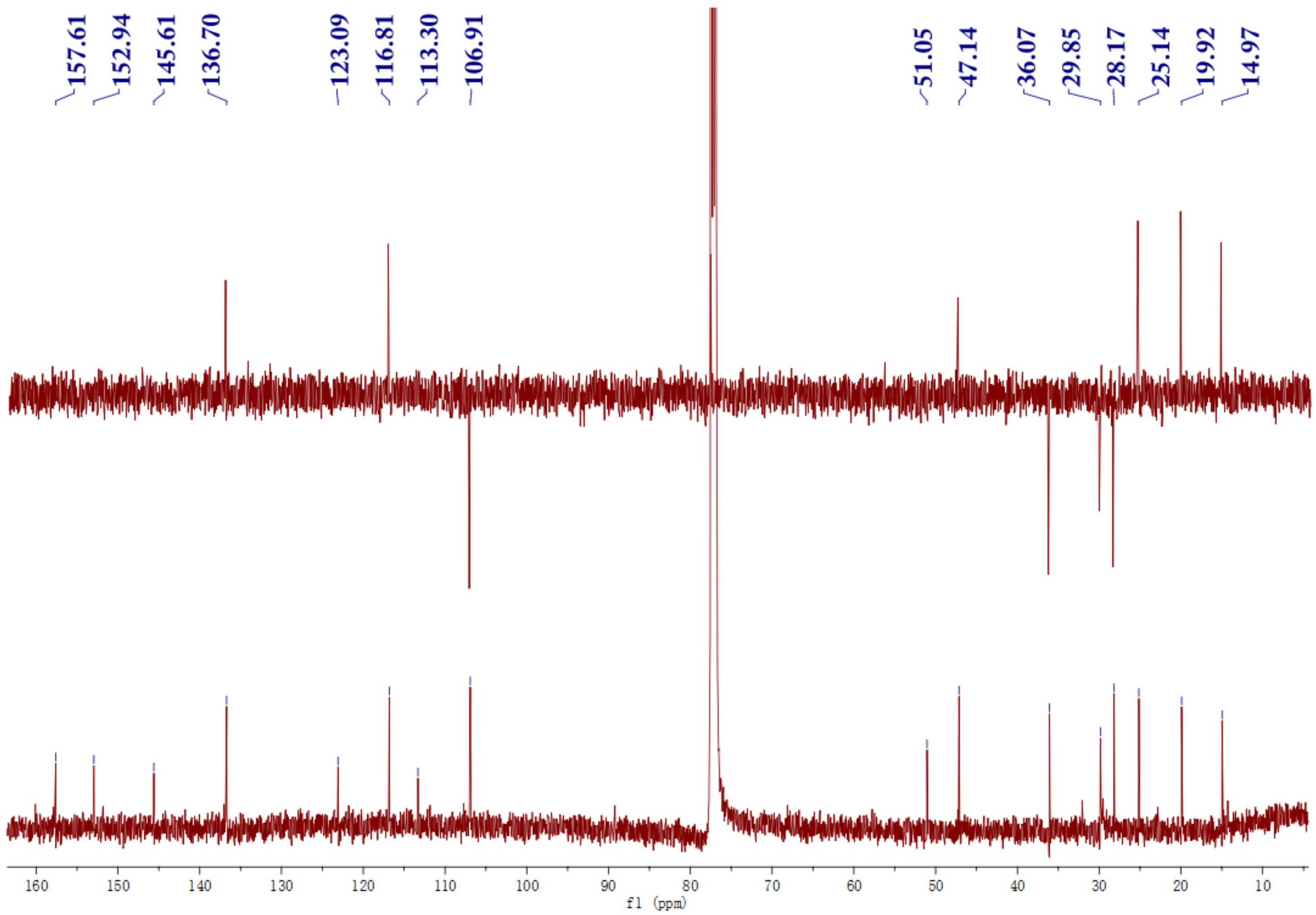

Figure S11: ^{1}H,^{1}H COSY spectrum (500 MHz, $CDCl_3$) of **2**.

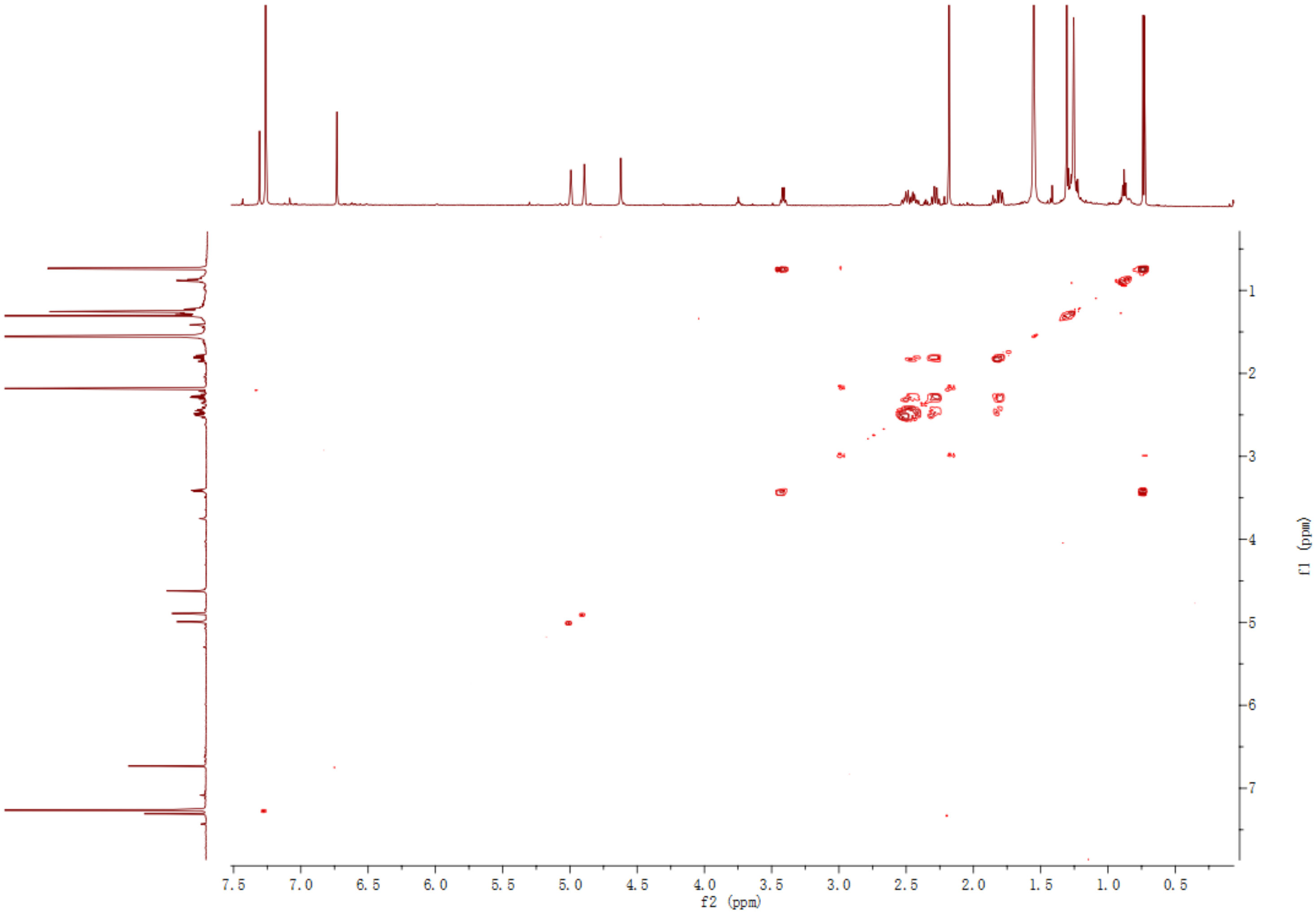

Figure S12: HMQC spectrum (500 MHz, $CDCl_3$) of **2**.

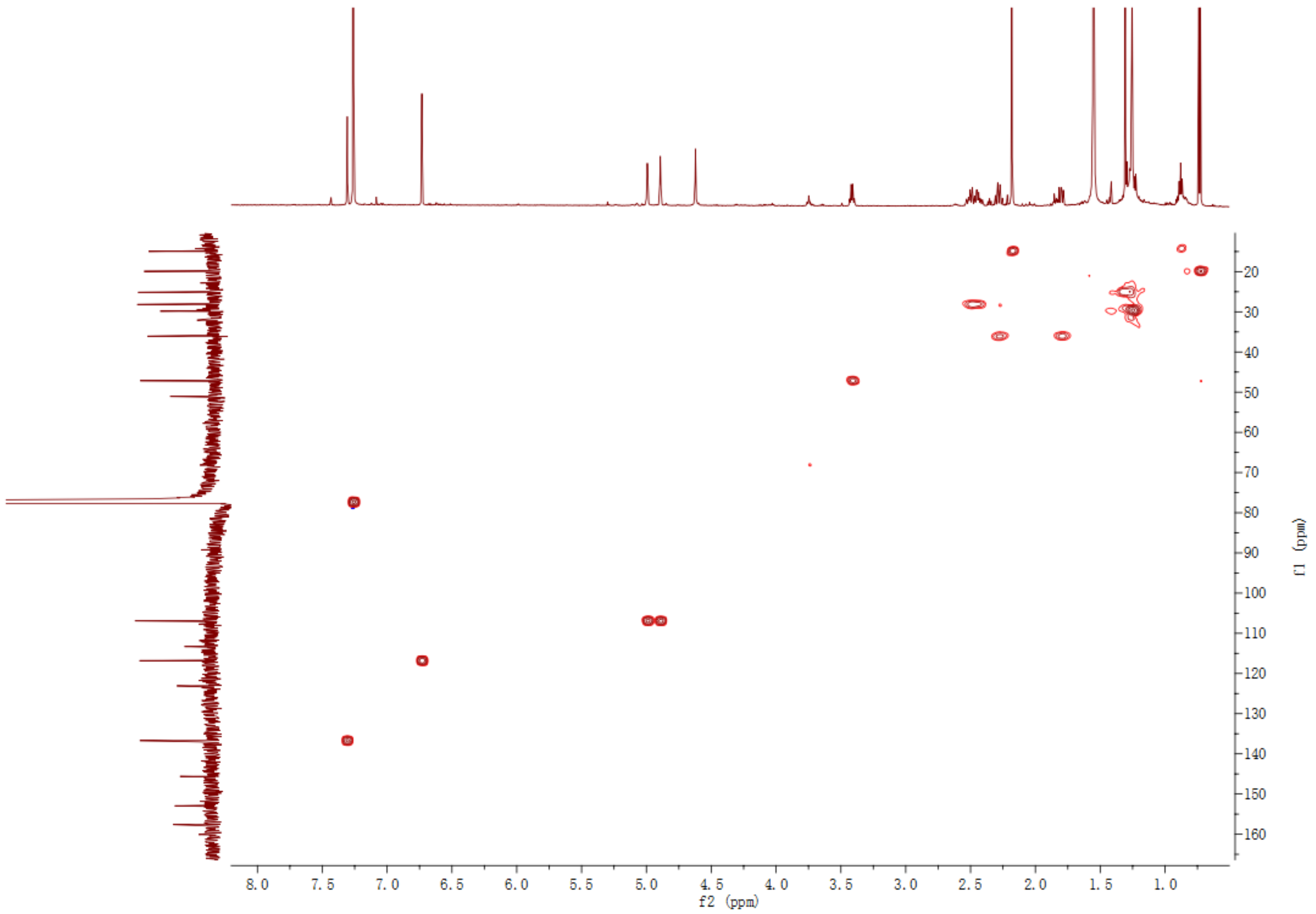

Figure S13: HMBC spectrum (500 MHz, $CDCl_3$) of **2**.

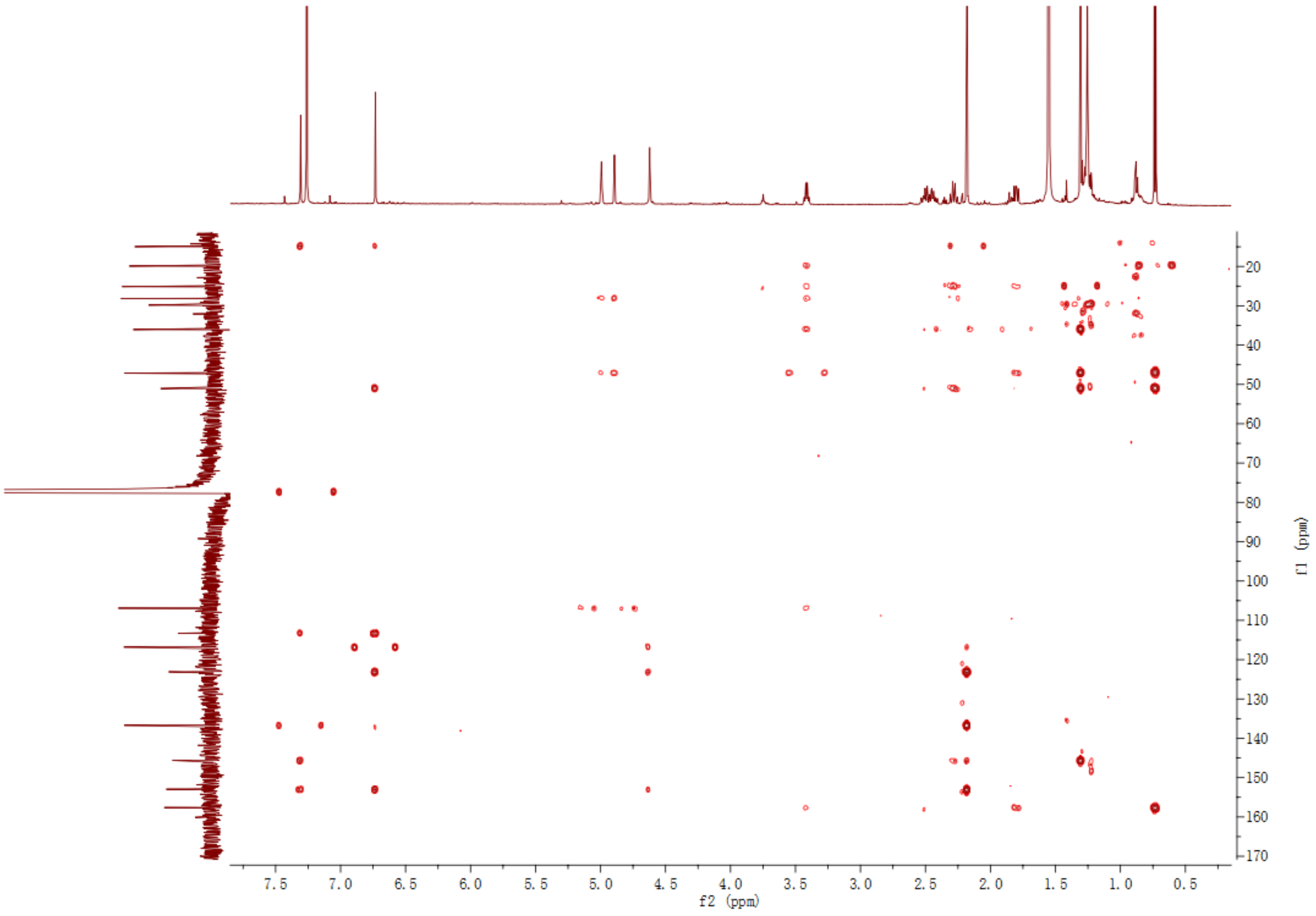

Figure S14: NOESY spectrum (500 MHz, $CDCl_3$) of **2**.

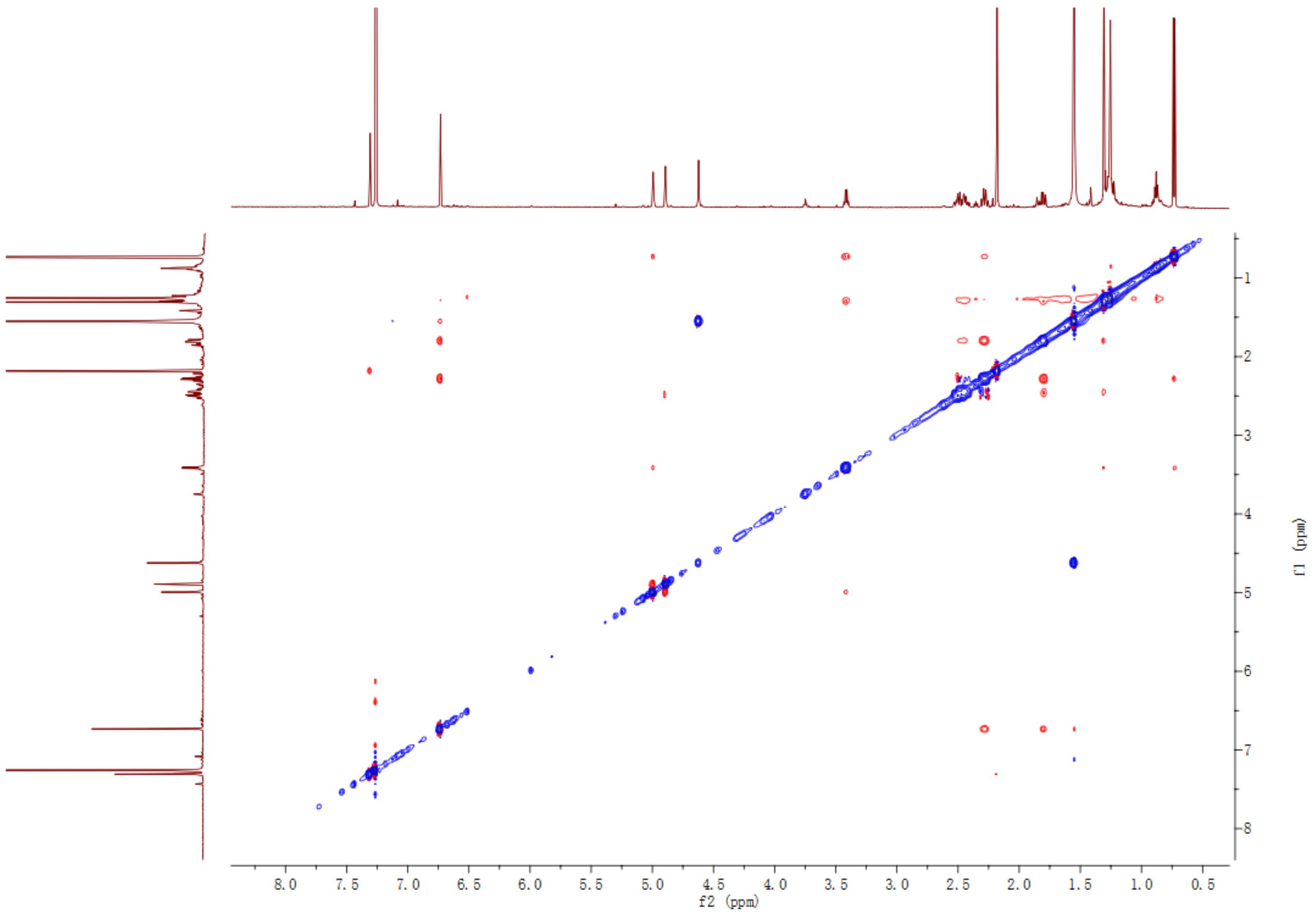

Figure S15: IR spectrum of **2**.

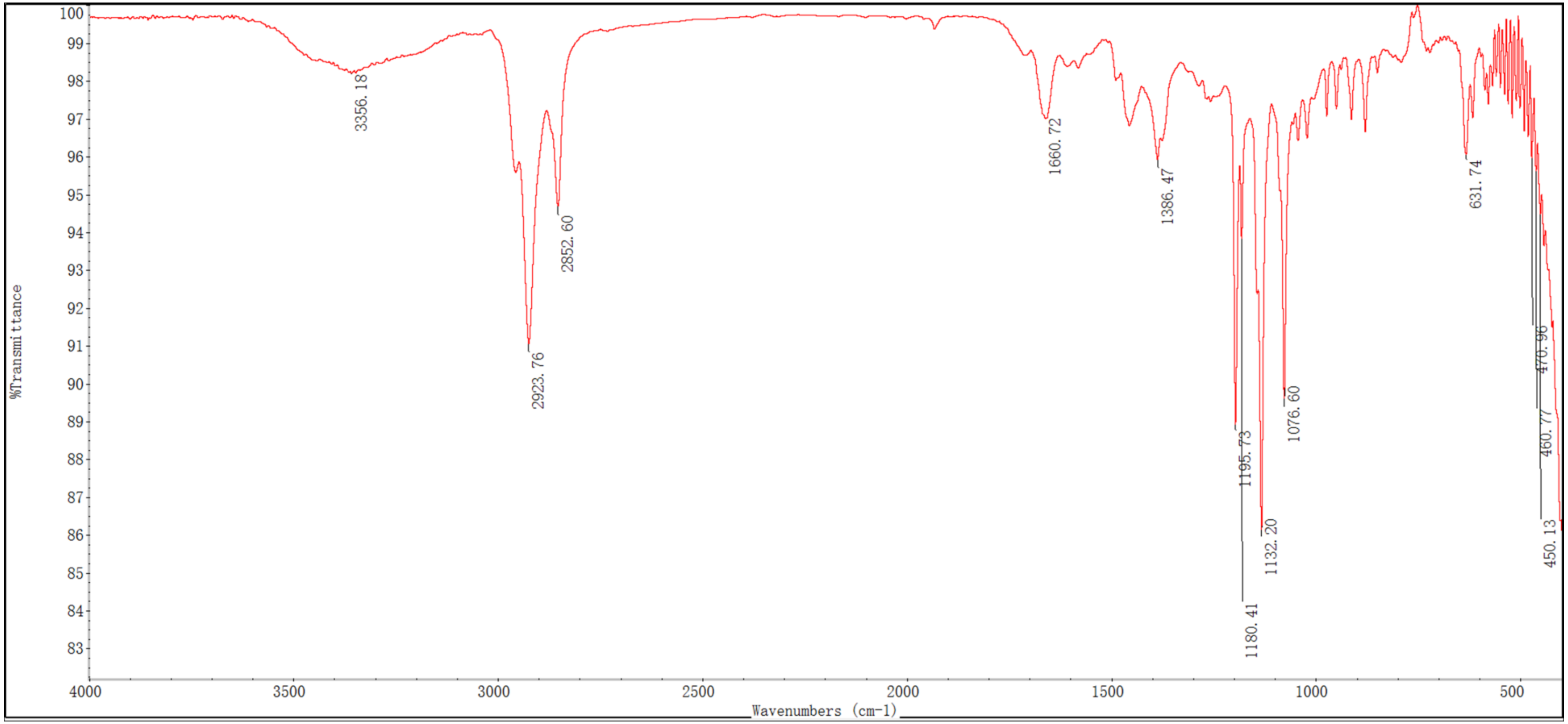

Figure S16: HR-ESIMS spectrum of **2**.

Elemental Composition Report

Page 1

Multiple Mass Analysis: 2 mass(es) processed

Tolerance = 20.0 PPM / DBE: min = -1.5, max = 50.0

Isotope cluster parameters: Separation = 1.0 Abundance = 1.0%

Monoisotopic Mass, Odd and Even Electron Ions

9 formula(e) evaluated with 1 results within limits (up to 20 closest results for each mass)

SIMM-Mass Spec Q-Tof Ultima 30-Sep-2015

D4g-2

151297-1 151 (2.855) AM (Cen,4, 80.00, Ht,9000.0,316.95,0.70); Sm (SG, 2x1.00); Cm (101:159) TOF MS ES- 1.49e3

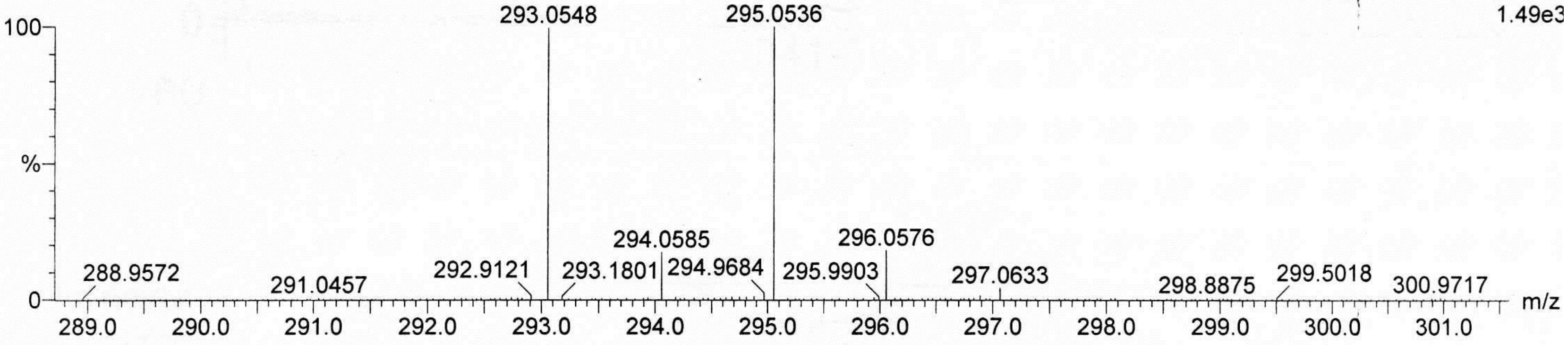

Minimum:	50.00				-1.5		
Maximum:	100.00		200.0	20.0	50.0		

Mass	RA	Calc. Mass	mDa	PPM	DBE	Score	Formula
293.0548	99.46	293.0541	0.7	2.4	6.5	1	C15 H18 O Br
295.0536	100.00	---					

Figure S17: ^{1}H NMR spectrum (500 MHz, $CDCl_3$) of **3**.

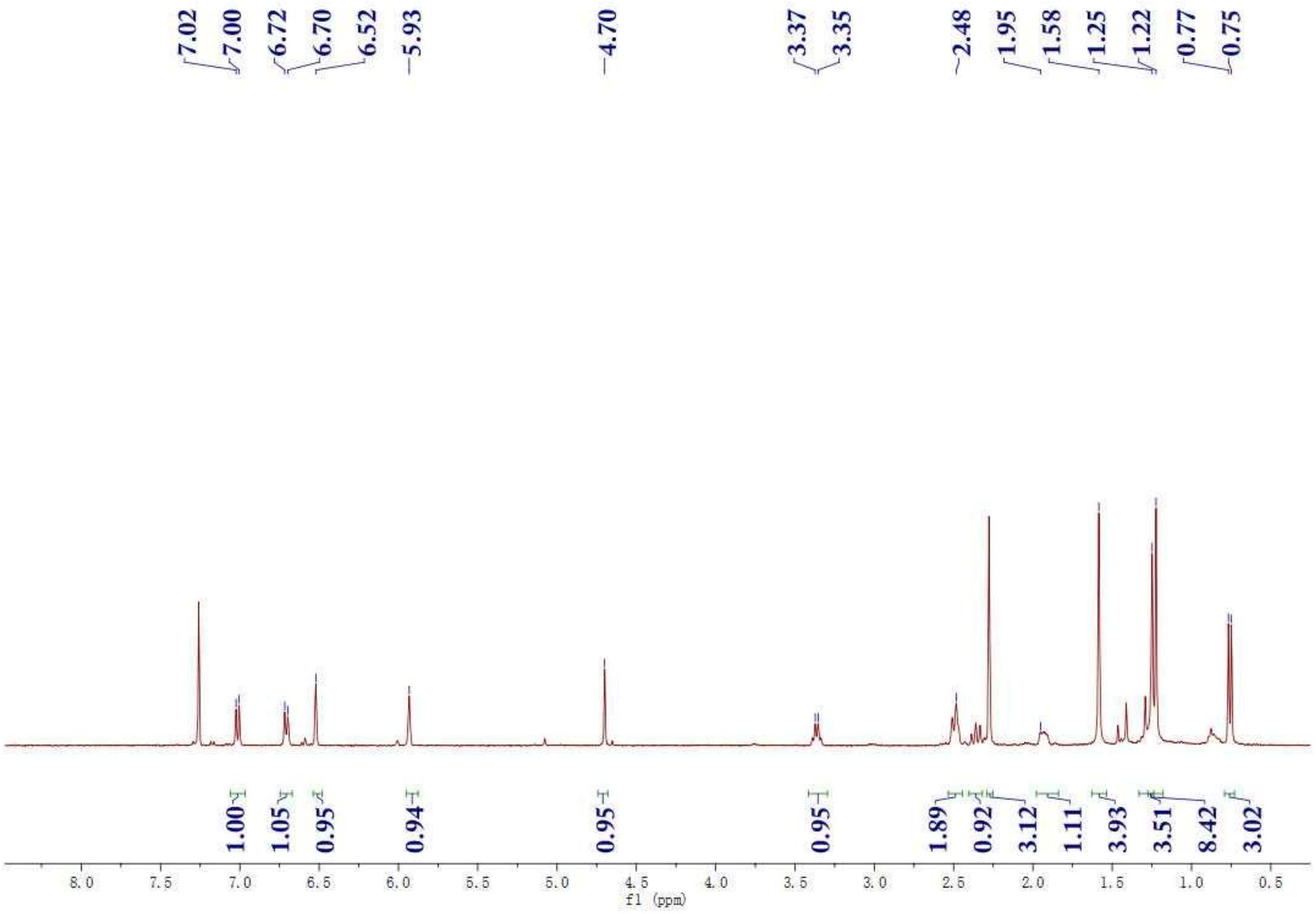

Figure S18: ^{13}C NMR spectrum (125 MHz, $CDCl_3$) of **3**.

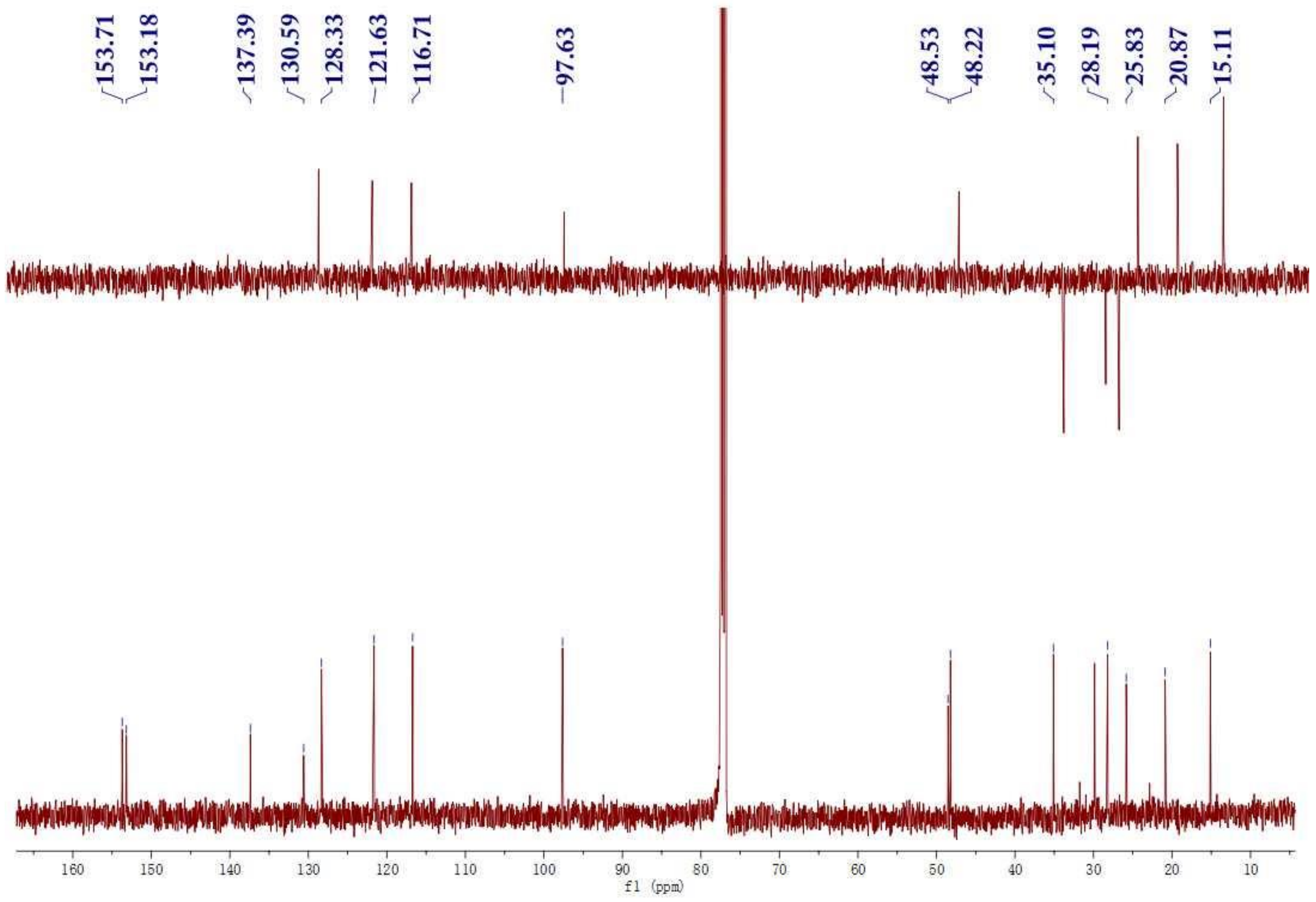

Figure S19: $^{1}H,^{1}H$ COSY spectrum (500 MHz, $CDCl_3$) of **3**.

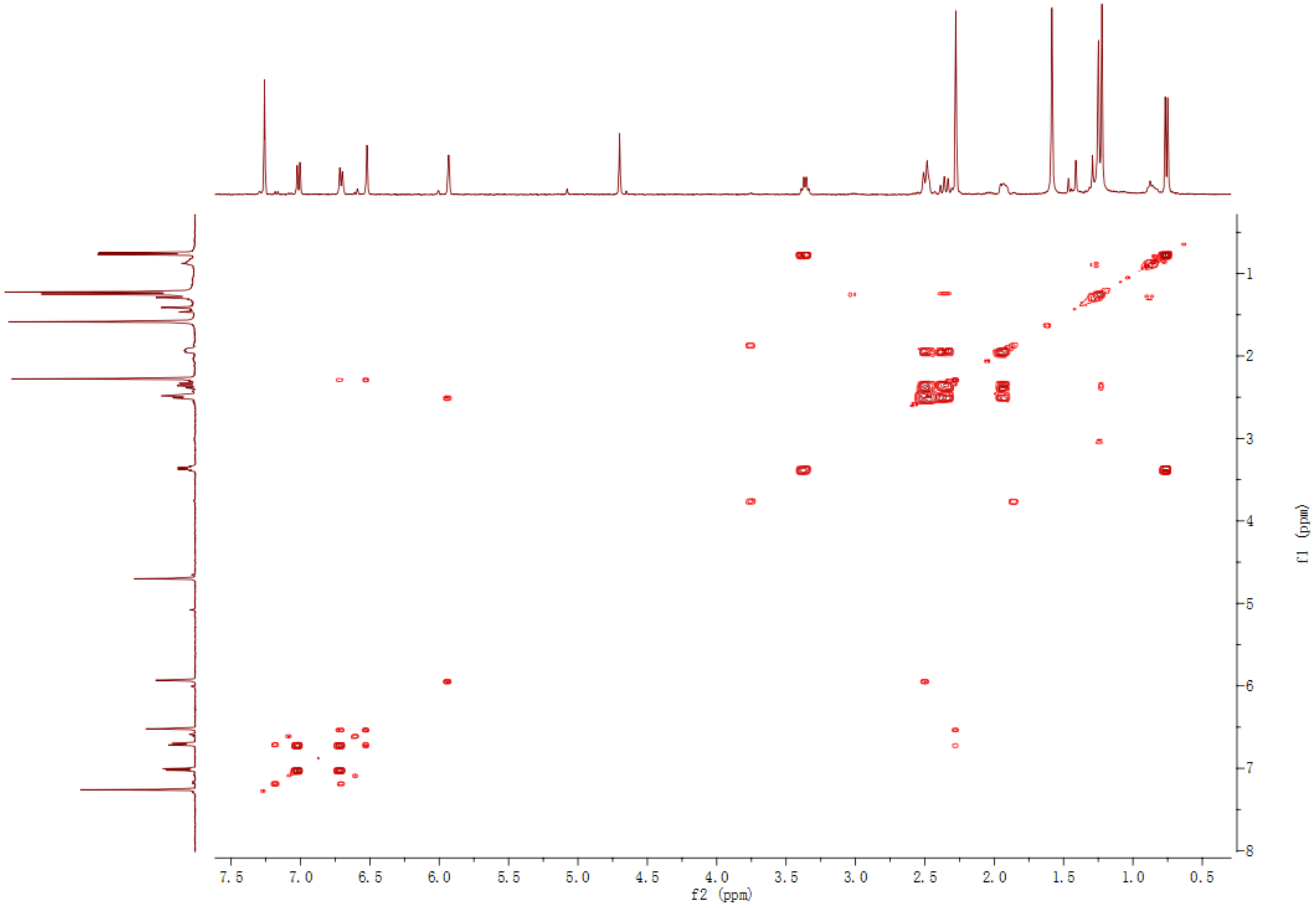

Figure S20: HMQC spectrum (500 MHz, $CDCl_3$) of **3**.

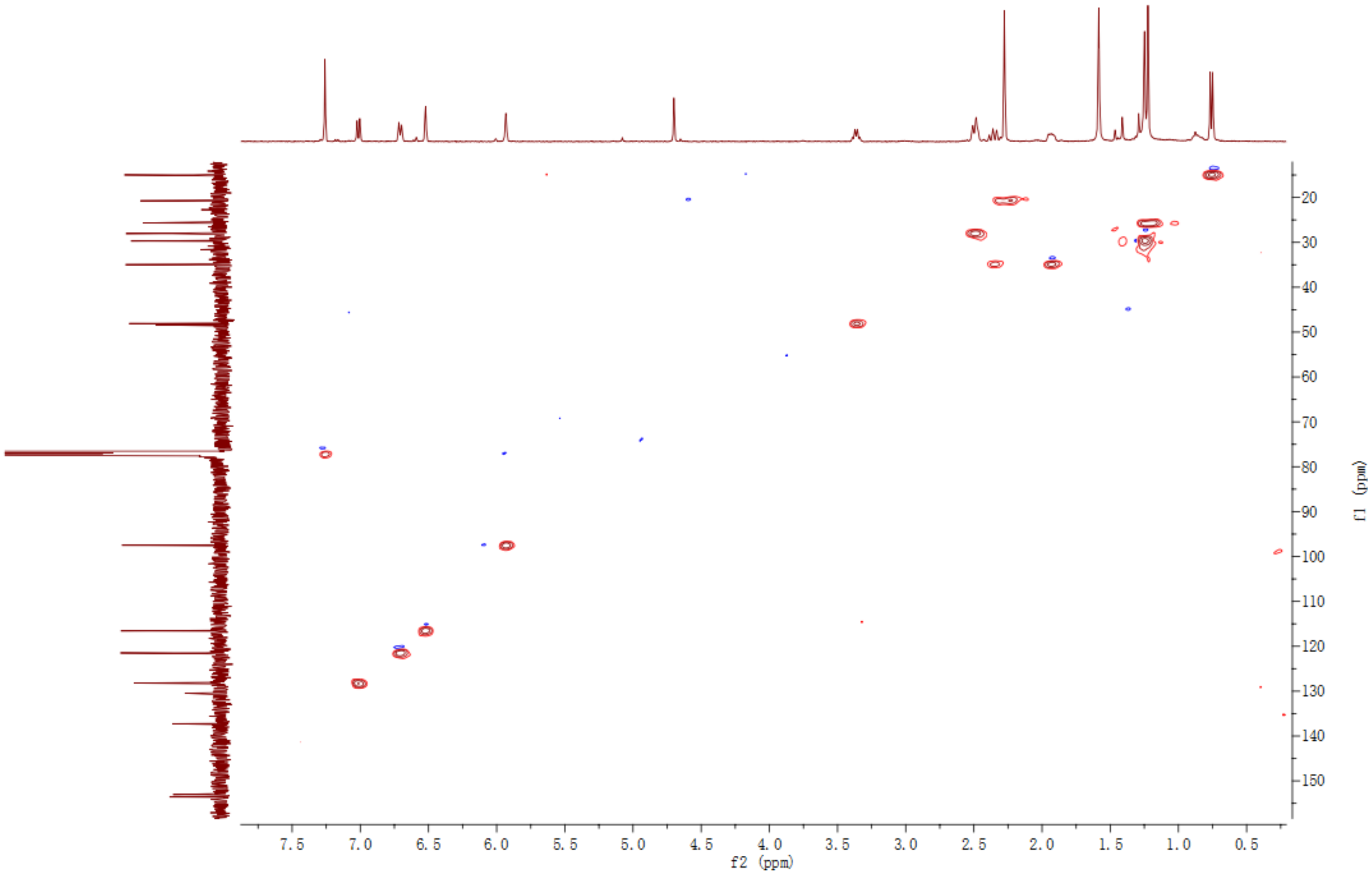

Figure S21: HMBC spectrum (500 MHz, $CDCl_3$) of **3**.

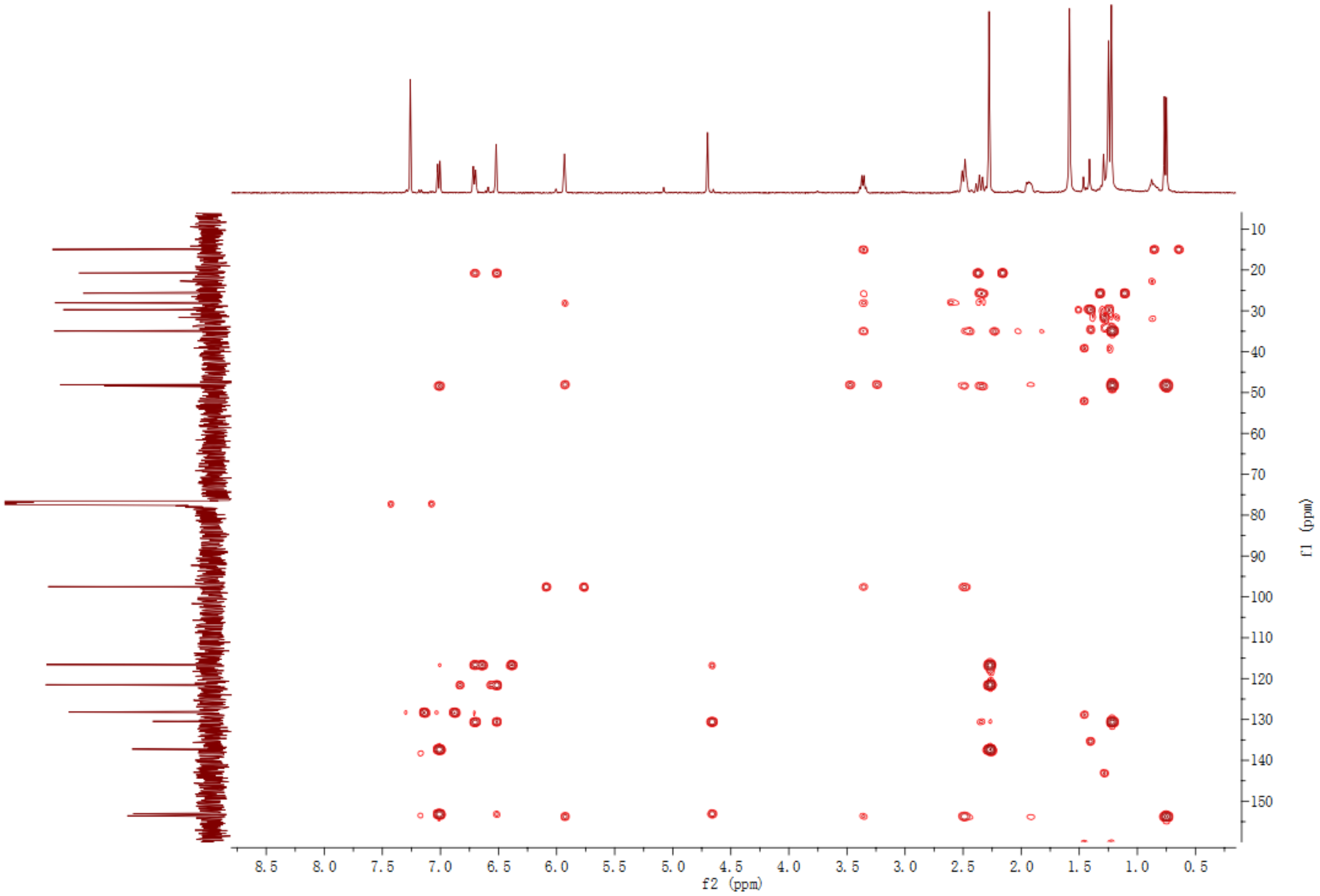

Figure S22: NOESY spectrum (500 MHz, $CDCl_3$) of **3**.

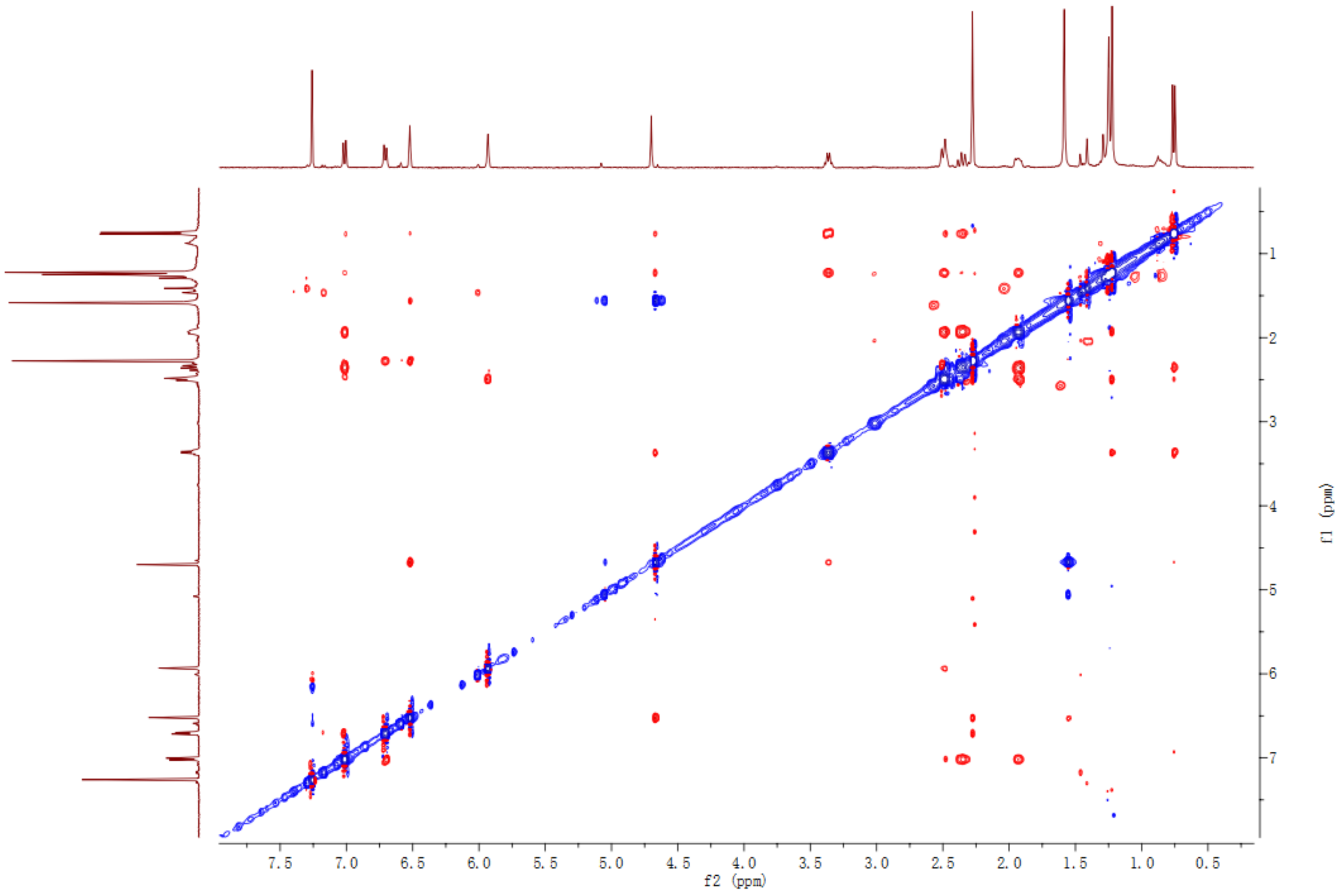

Figure S23: IR spectrum of **3**.

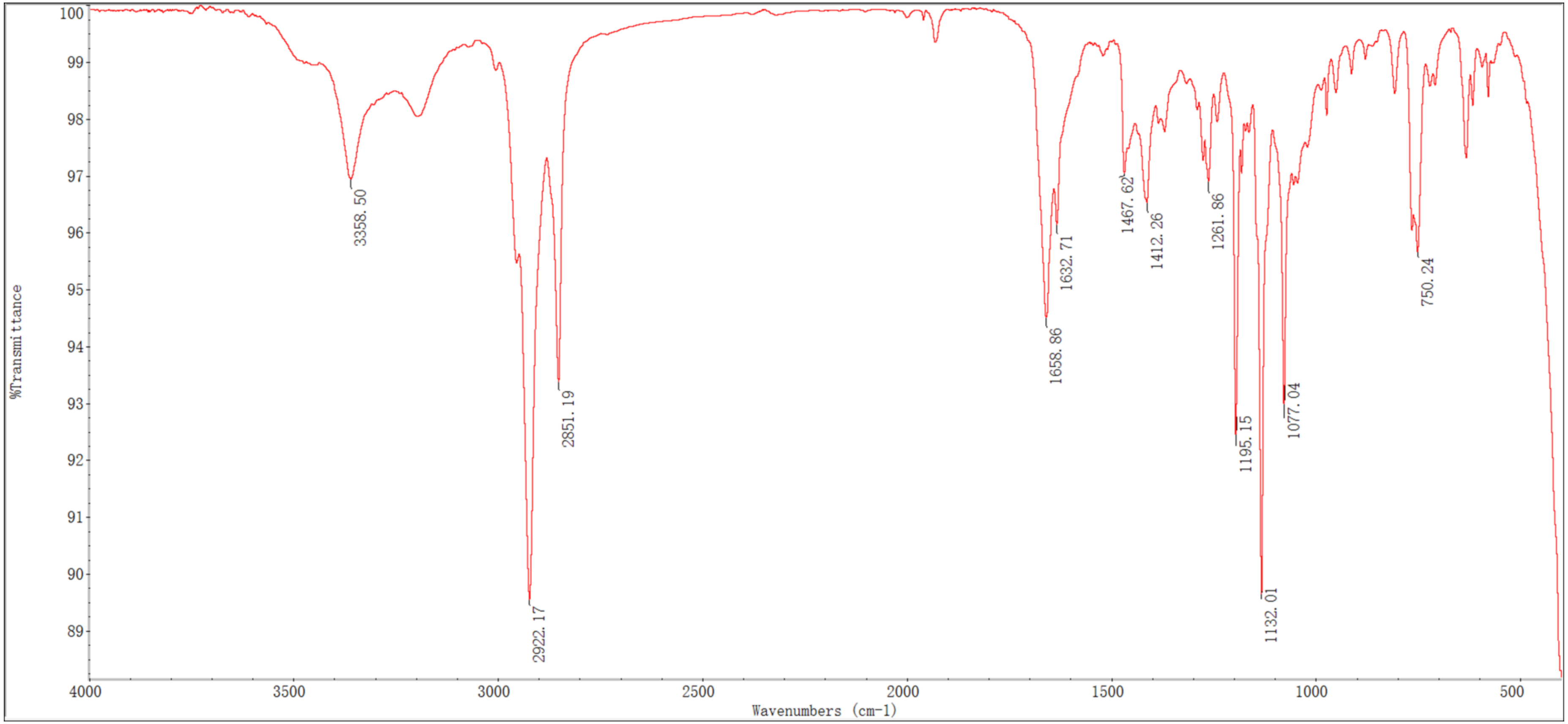

Figure S24: HR-EIMS spectrum of **3**.

```
LIST:  h150453-c2                        26-Sep-15  Elapse:  01:38.4      15
Samp:  c4a2                                         Start :  17:37:12     18
Comm:  Finnigan/MAT95/70eV/R:10000
Mode:  EI +VE +LMR  BSCAN (EXP) UP HR NRM           Study :  S/N: PT200712-01-01
Oper:  SIMM.CAS      Client:  S/N: PT001263         Inlet :
Limt:  (   0 )     .    .  .
    :  ( 404 )  C16.H100.Br.O2
Peak:  1000.00  mmu     R+D:  -2.0 > 60.0
Data:  CMASS : converted
```

Mass		Intensity (4277)	%RA	%RIC	Delta (mmu)	R+D	Composition
55.06891	*	5569	2.60	0.03			
57.08490	*	5806	2.71	0.03			
67.06923	*	5450	2.55	0.03			
69.07027	*	6043	2.83	0.03	0.2	1.5	C5.H9
77.03876	*	10190	4.76	0.05	0.4	4.5	C6.H5
79.05401	*	6161	2.88	0.03	0.8	3.5	C6.H7
81.06935	*	8650	4.04	0.04	1.1	2.5	C6.H9
91.05108	*	13626	6.37	0.07			
92.99269	*	93137	43.55	0.45			
95.08308	*	4739	2.22	0.02	3.0	2.5	C7.H11
105.0727	*	11731	5.48	0.06	-2.3	4.5	C8.H9
107.0905	*	19670	9.20	0.10			
111.9990	*	28675	13.41	0.14			
115.0565	*	9361	4.38	0.05	-1.7	6.5	C9.H7
121.0658	*	100484	46.98	0.49	-0.4	4.5	C8.H9.O
122.0693	*	4384	2.05	0.02			
128.0619	*	5569	2.60	0.03	0.7	7.0	C10.H8
135.0821	*	24410	11.41	0.12	-1.1	4.5	C9.H11.O
145.0667	*	15522	7.26	0.08	-1.4	6.5	C10.H9.O
146.9841	*	4502	2.10	0.02			
147.0798	*	4384	2.05	0.02	1.2	5.5	C10.H11.O
149.0249	*	7583	3.55	0.04			
158.0730	*	4502	2.10	0.02	0.2	7.0	C11.H10.O
159.0809	*	38748	18.12	0.19	0.1	6.5	C11.H11.O
160.0870	*	9005	4.21	0.04	1.8	6.0	C11.H12.O
171.0823	*	4621	2.16	0.02	-1.3	7.5	C12.H11.O
173.0982	*	20144	9.42	0.10	-1.5	6.5	C12.H13.O
185.0952	*	15167	7.09	0.07	1.4	7.5	C13.H13.O
186.1021	*	6517	3.05	0.03	2.4	7.0	C13.H14.O
187.1103	*	8413	3.93	0.04	2.0	6.5	C13.H15.O
191.1435	*	8768	4.10	0.04	0.1	4.5	C13.H19.O
199.1113	*	17655	8.25	0.09	1.0	7.5	C14.H15.O
200.1184	*	14930	6.98	0.07	1.7	7.0	C14.H16.O
207.0335	*	14337	6.70	0.07			
214.1352	*	7228	3.38	0.04	0.5	7.0	C15.H18.O
215.1415	*	213884	100.00	1.04	2.1	6.5	C15.H19.O
216.1460	*	23225	10.86	0.11			
279.0407	*	8768	4.10	0.04	-2.2	6.5	C14.H16.Br.O
281.0369	*	11968	5.60	0.06			
294.0617	*	6754	3.16	0.03	0.2	6.0	C15.H19.Br.O
296.0594	*	7109	3.32	0.03			

Figure S25: ^{1}H NMR spectrum (500 MHz, $CDCl_3$) of **4**.

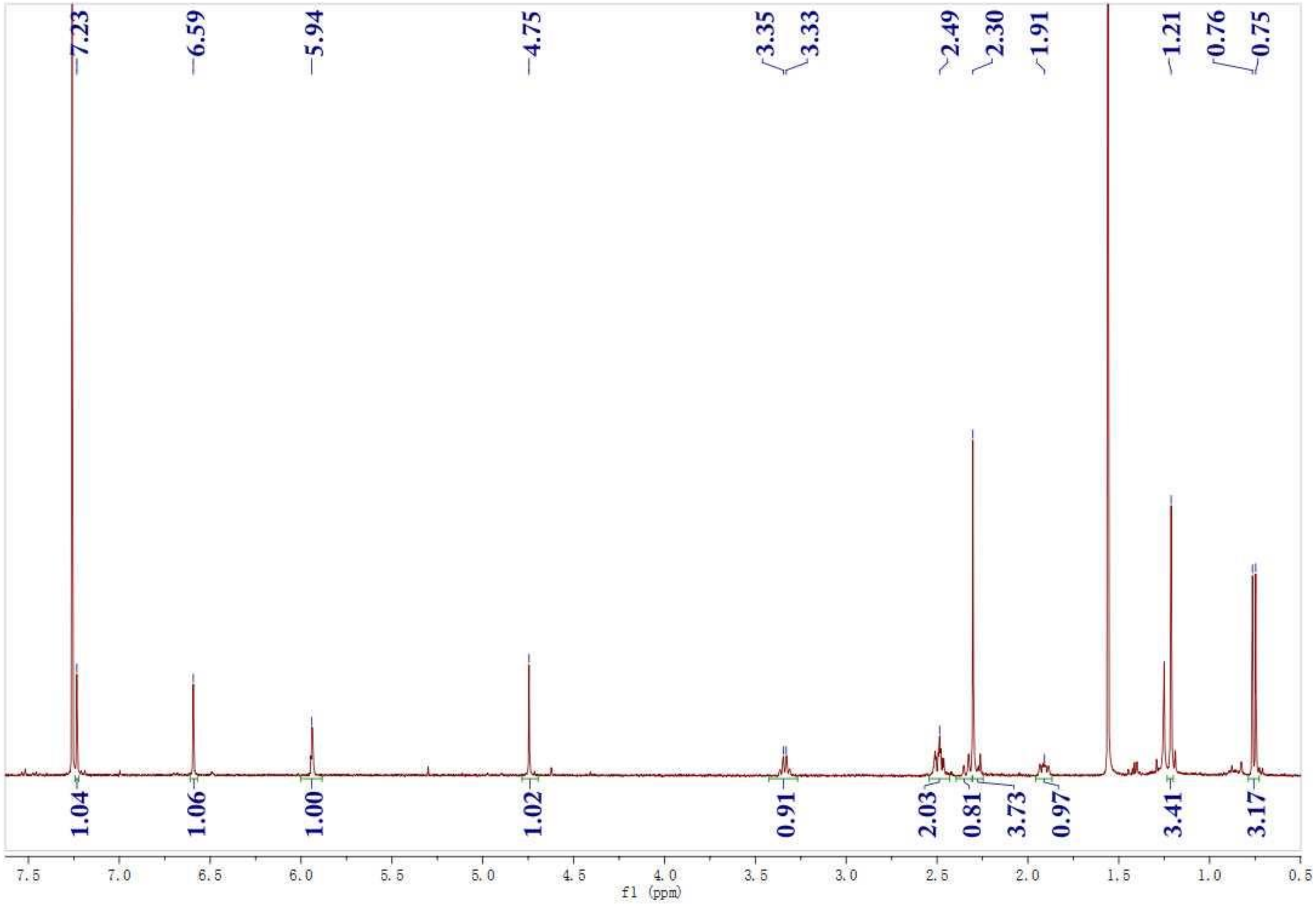

Figure S26: ^{13}C NMR spectrum (125 MHz, $CDCl_3$) of **4**.

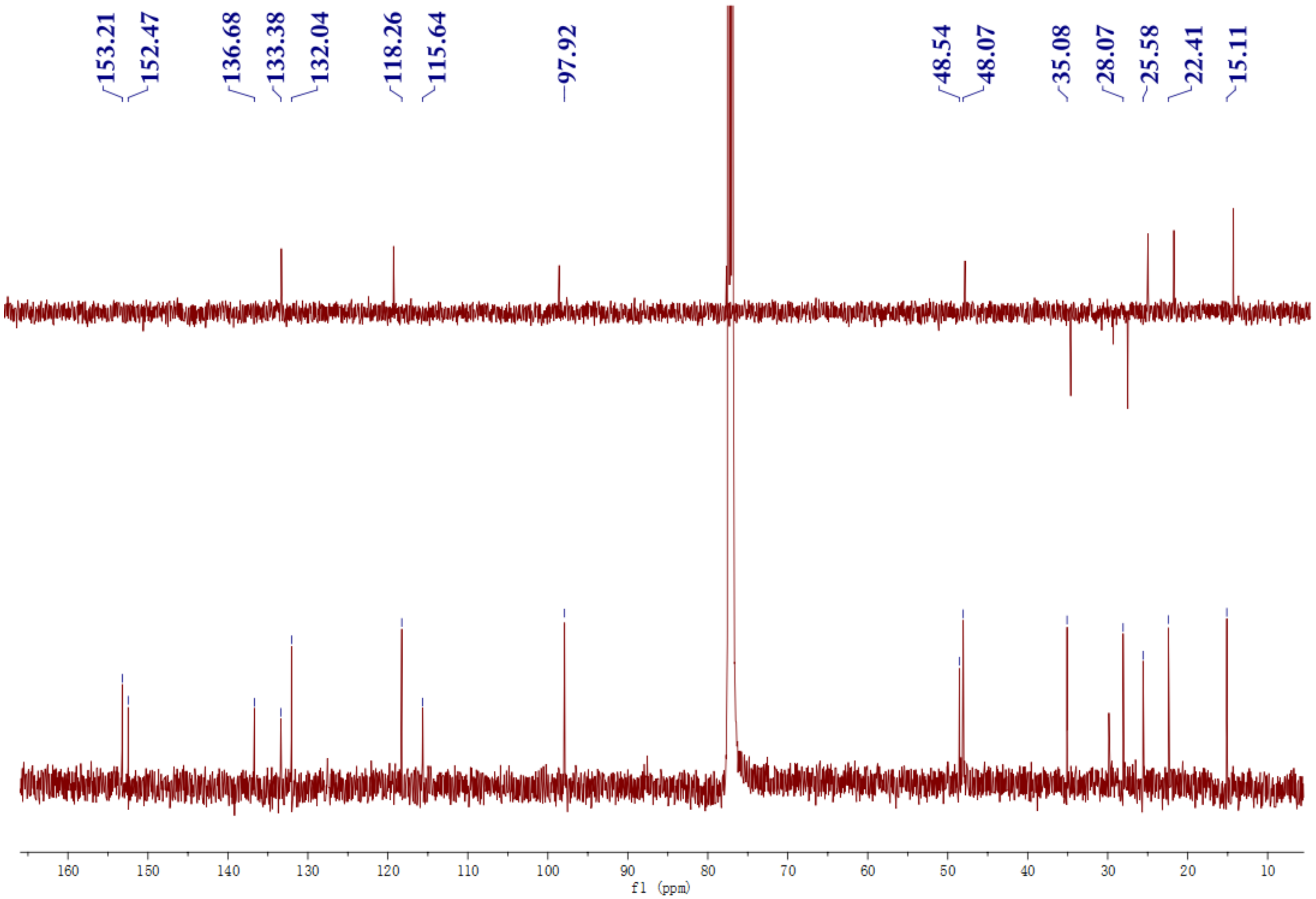

Figure S27: ^{1}H,^{1}H COSY spectrum (500 MHz, $CDCl_3$) of **4**.

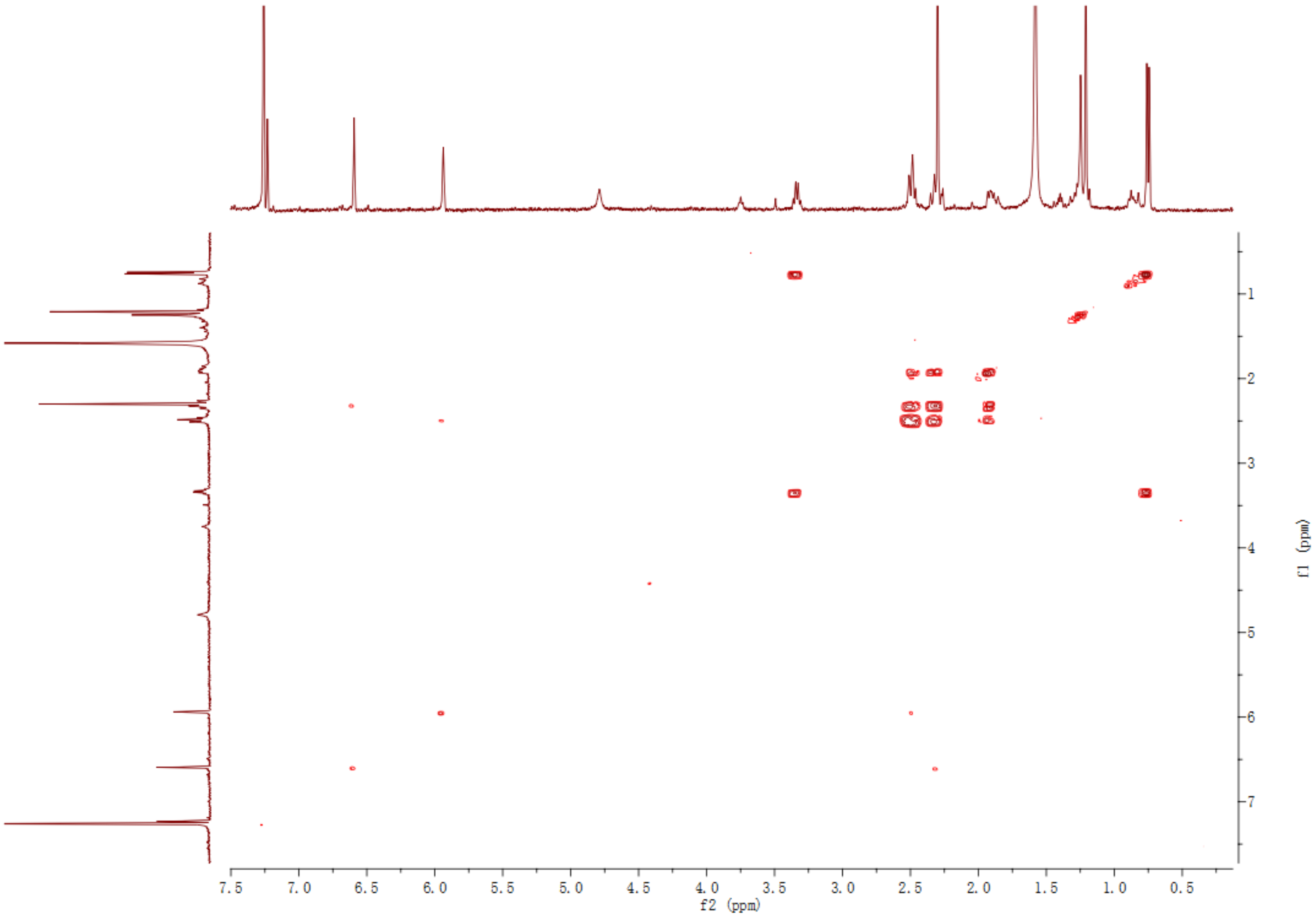

Figure S28: HMQC spectrum (500 MHz, $CDCl_3$) of **4**.

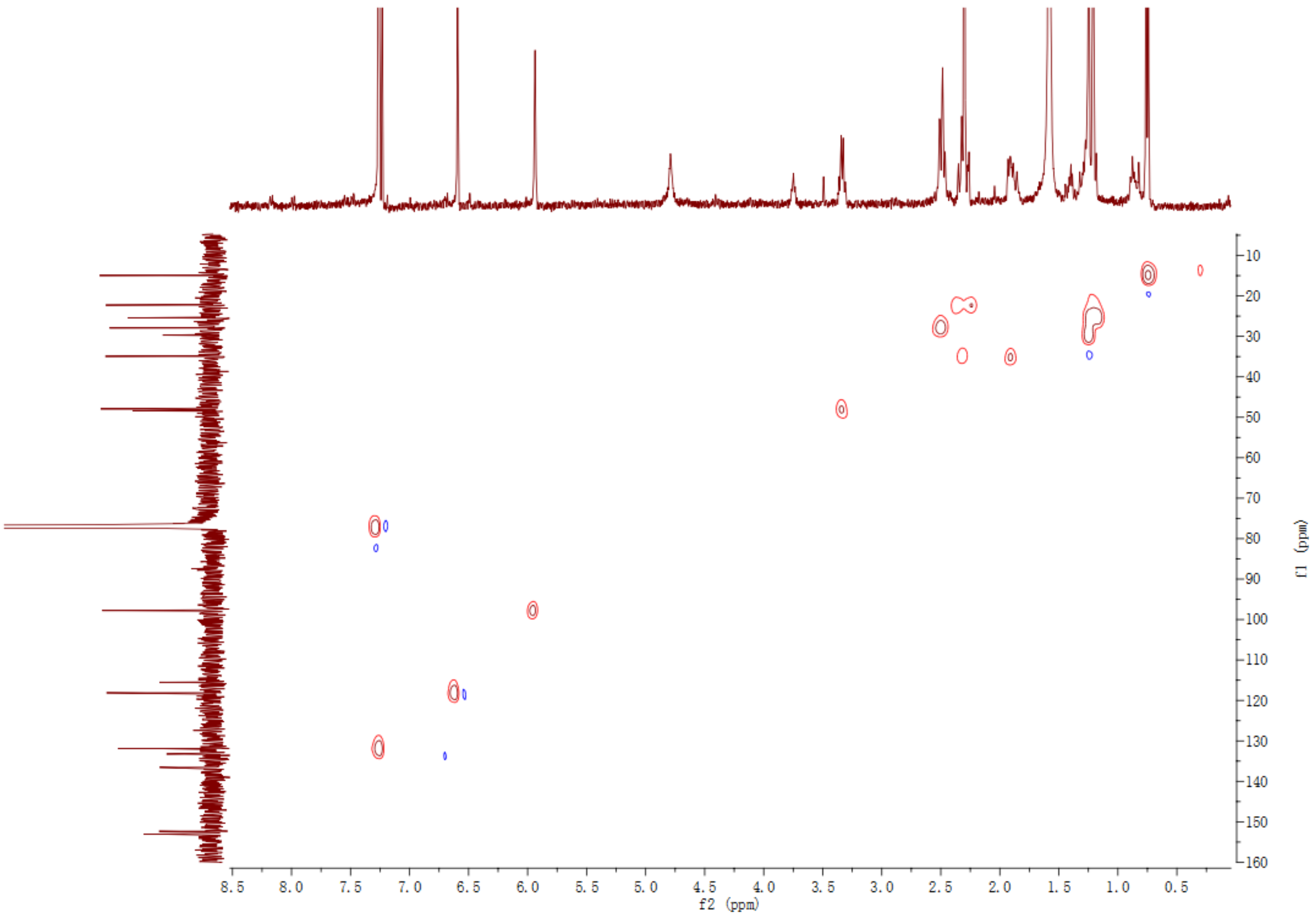

Figure S29: HMBC spectrum (500 MHz, $CDCl_3$) of **4**.

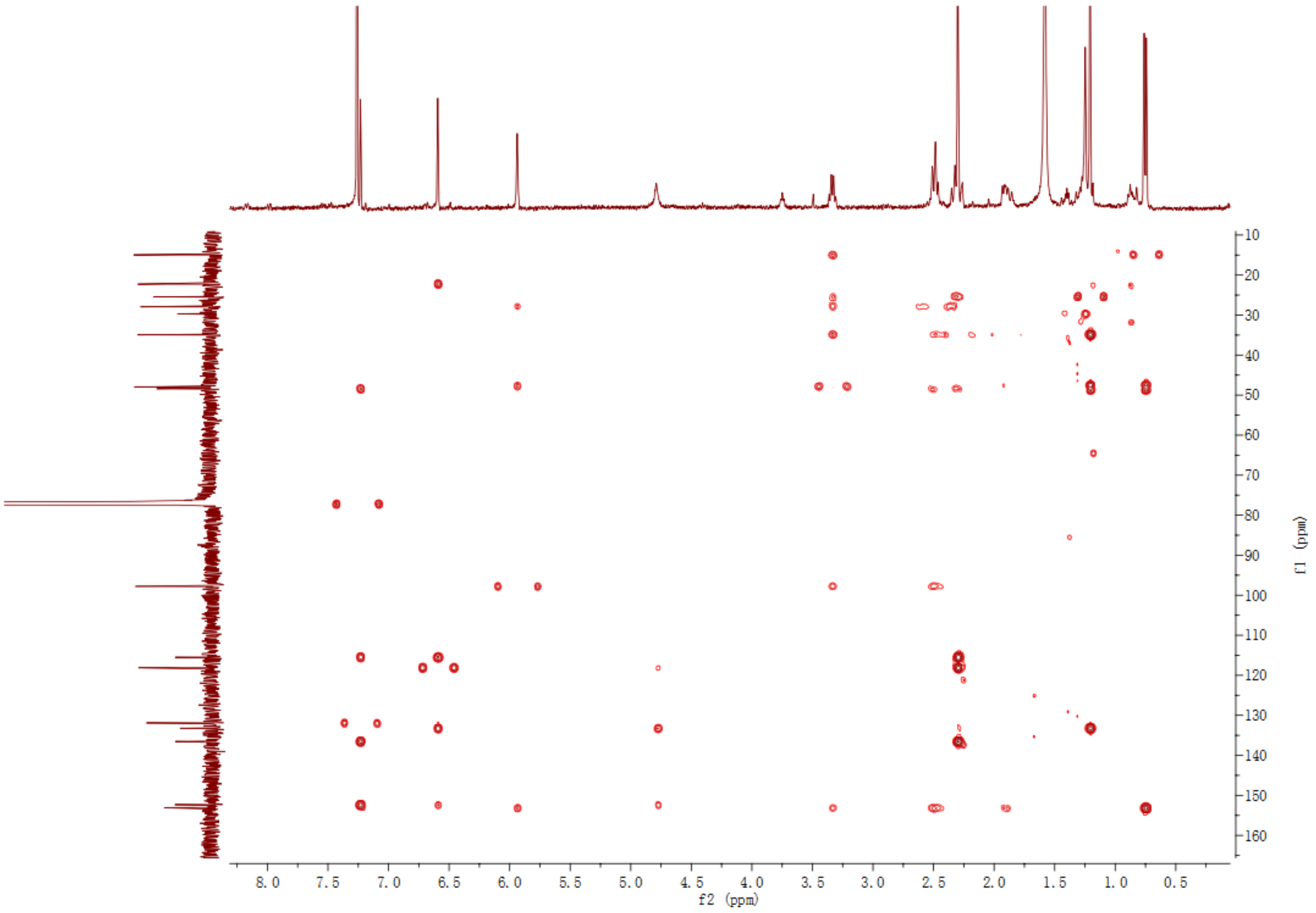

Figure S30: NOESY spectrum (500 MHz, $CDCl_3$) of **4**.

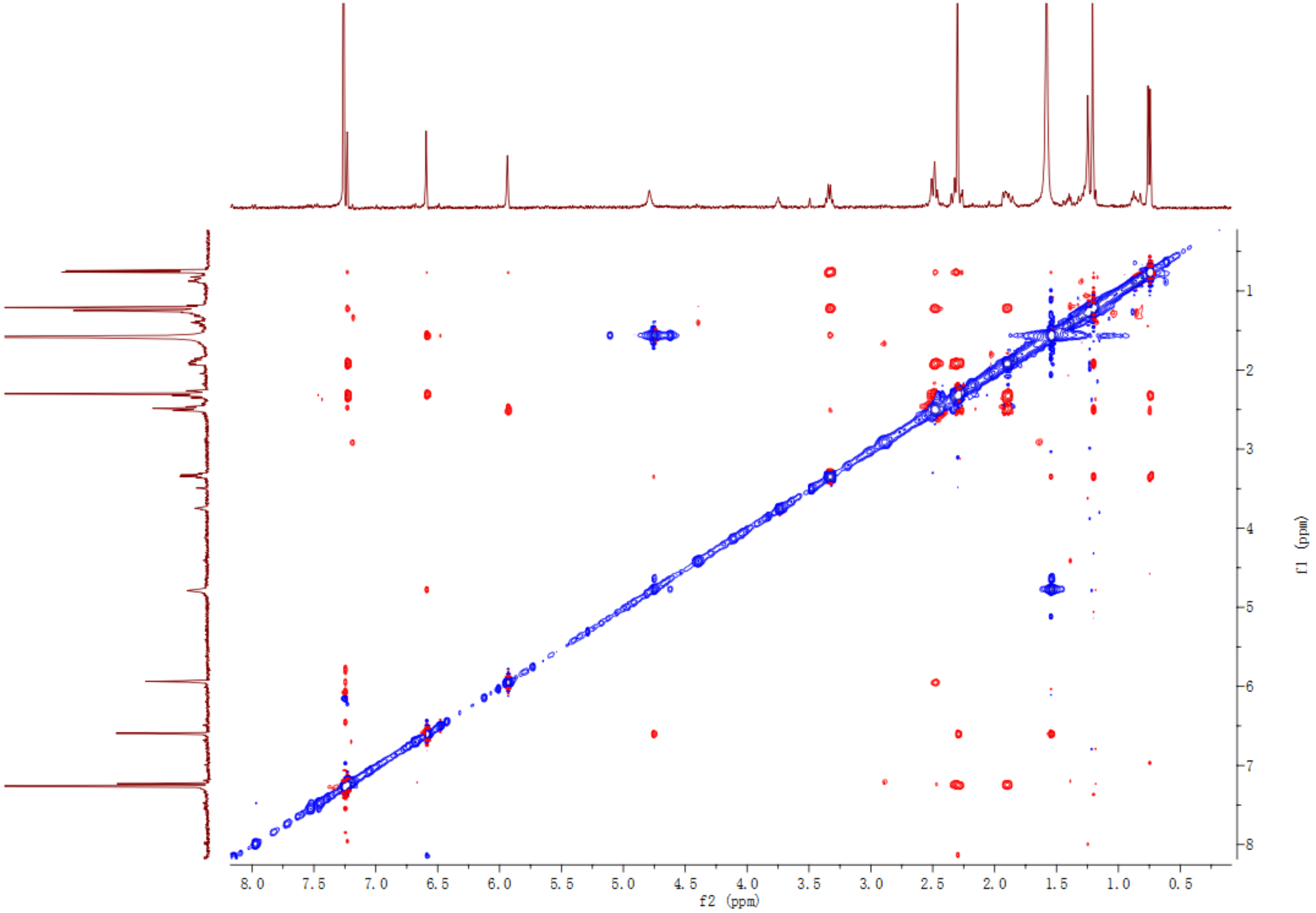

Figure S31: IR spectrum of **4**.

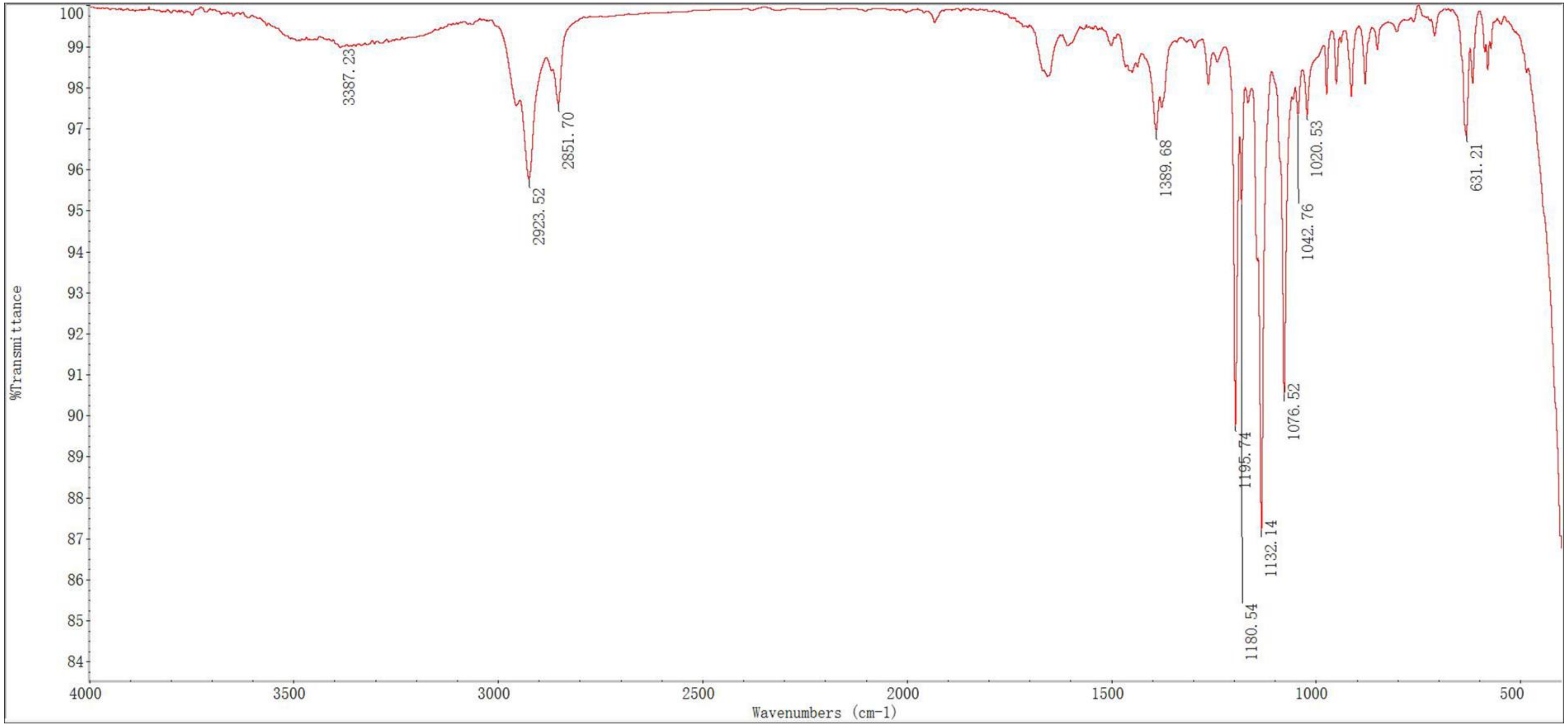

Figure S32: HR-ESIMS spectrum of **4**.

Elemental Composition Report

Multiple Mass Analysis: 3 mass(es) processed

Tolerance = 20.0 PPM / DBE: min = -1.5, max = 50.0

Isotope cluster parameters: Separation = 1.0 Abundance = 1.0%

Monoisotopic Mass, Odd and Even Electron Ions

19 formula(e) evaluated with 1 results within limits (up to 20 closest results for each mass)

SIMM-Mass Spec Q-Tof Ultima 30-Sep-2015

D4d

151298-1 98 (1.858) AM (Cen,4, 80.00, Ht,9000.0,384.93,0.70); Sm (SG, 2x1.00); Cm (98:120) TOF MS ES- 1.57e3

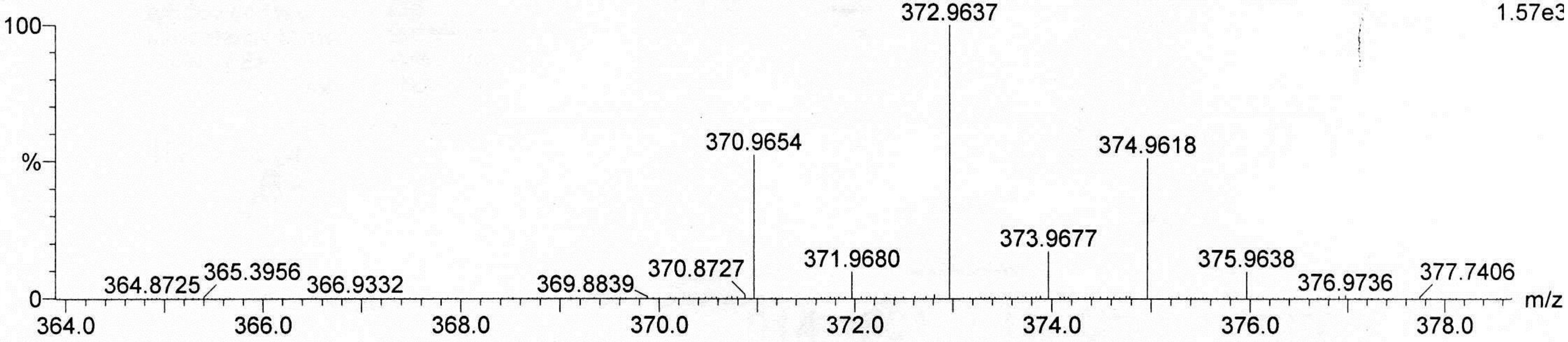

Minimum:	50.00				-1.5		
Maximum:	100.00		200.0	20.0	50.0		

Mass	RA	Calc. Mass	mDa	PPM	DBE	Score	Formula
370.9654	52.30	370.9646	0.8	2.1	6.5	1	C15 H17 O Br2
372.9637	100.00	---					
374.9618	51.26	---					

Figure S33: ^{1}H NMR spectrum (500 MHz, $CDCl_3$) of **6**.

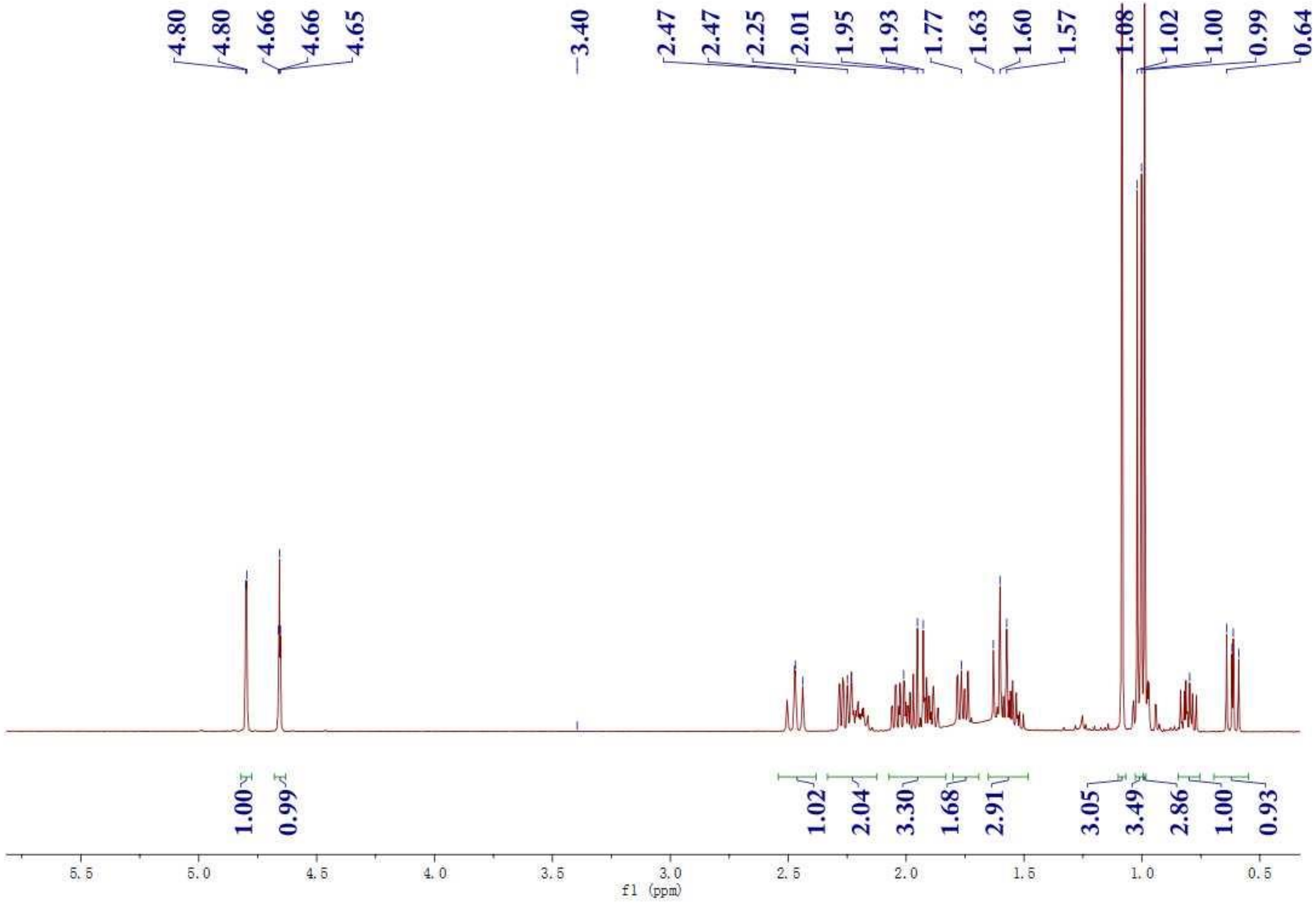

Figure S34: ^{13}C NMR spectrum (125 MHz, $CDCl_3$) of **6**.

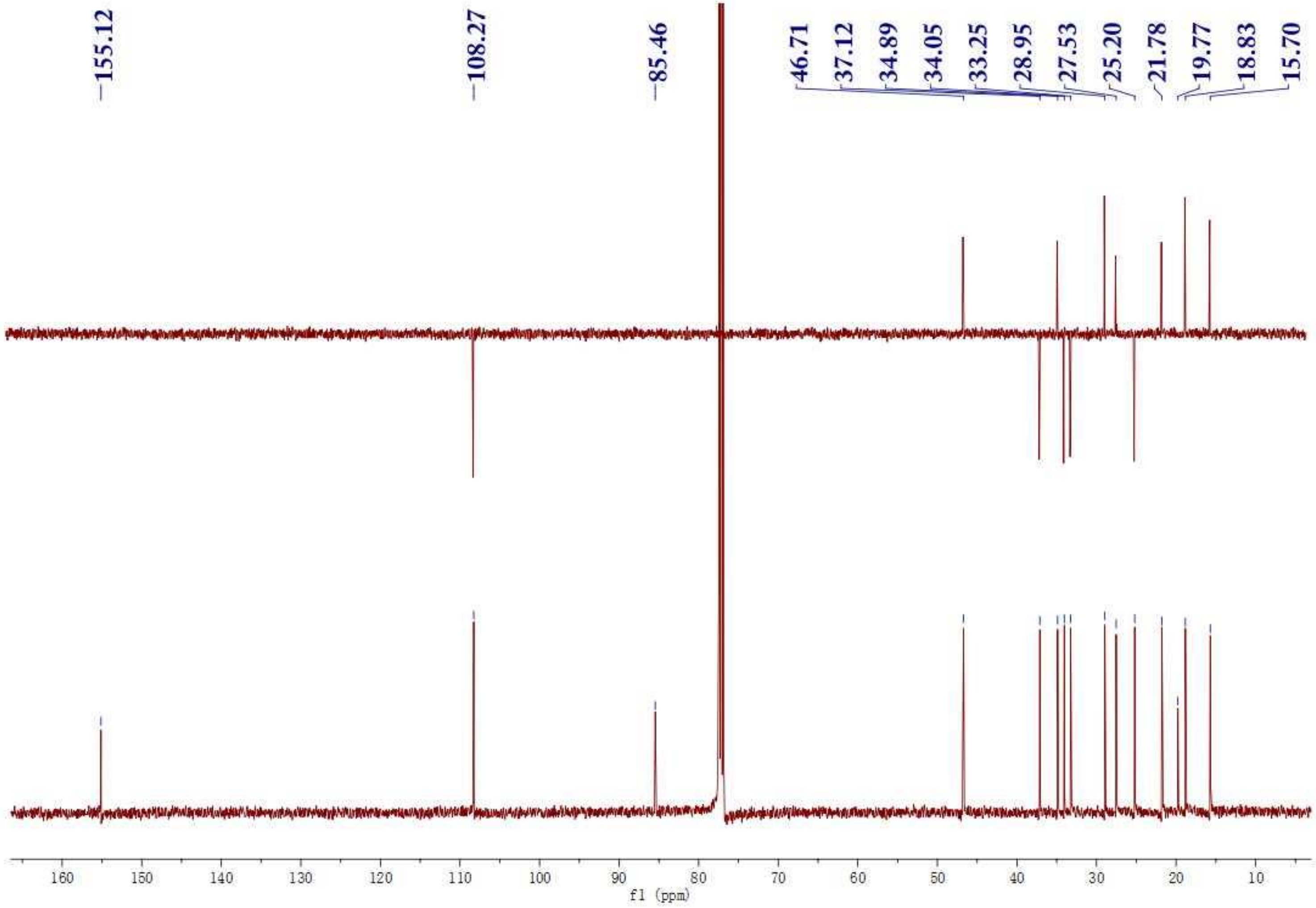

Figure S35: $^{1}H,^{1}H$ COSY spectrum (500 MHz, $CDCl_3$) of **6**.

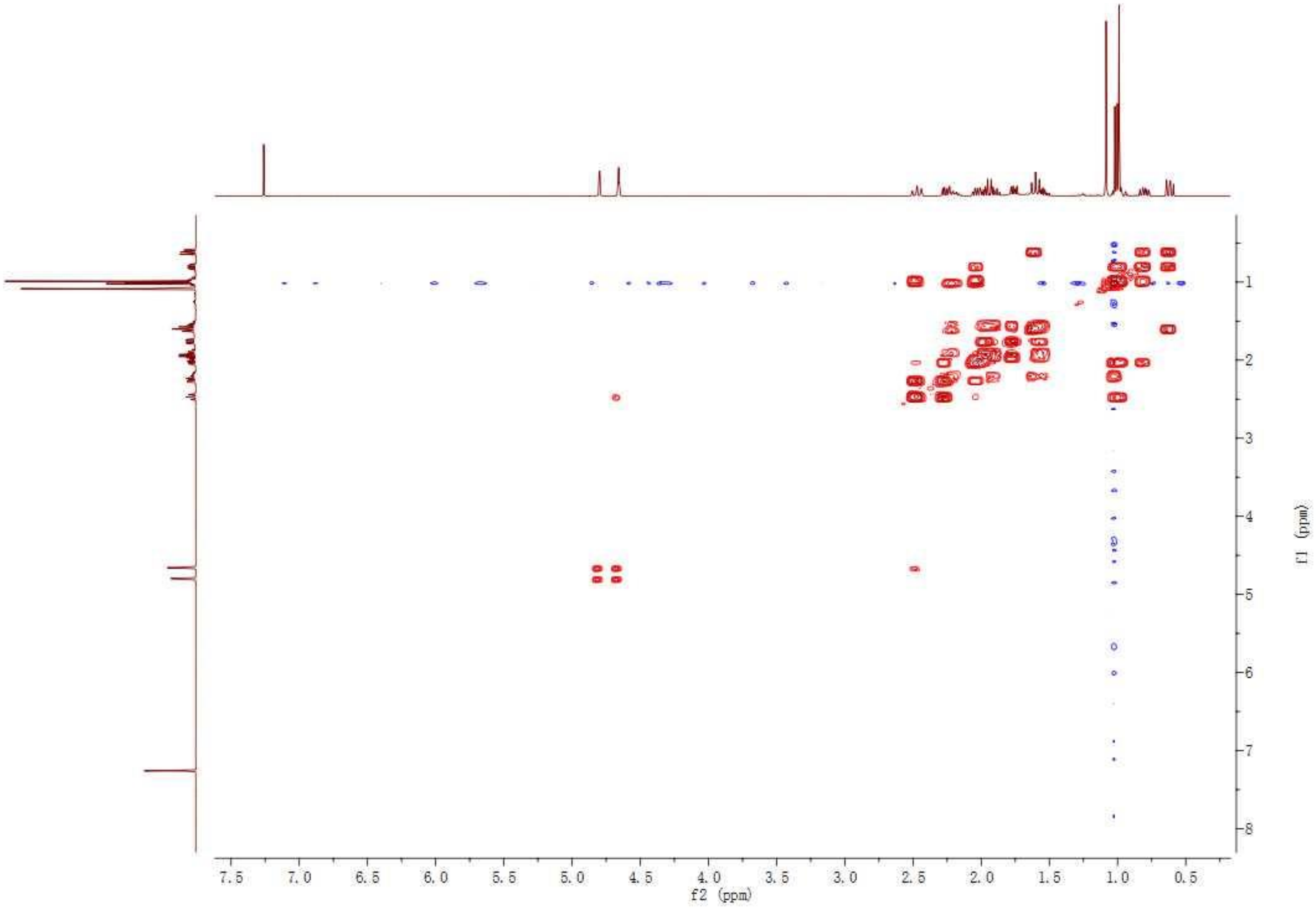

Figure S36: HMQC spectrum (500 MHz, $CDCl_3$) of **6**.

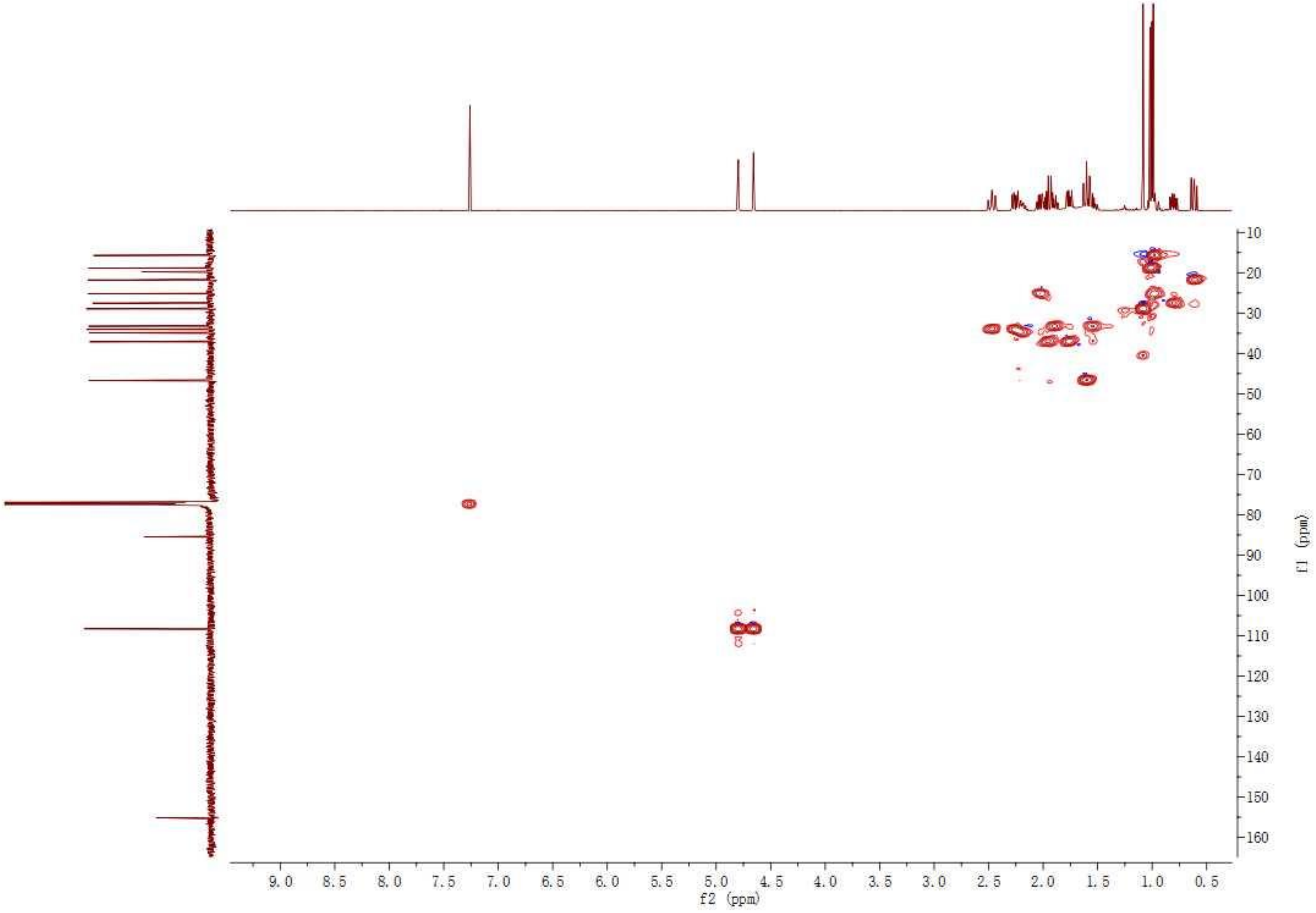

Figure S37: HMBC spectrum (500 MHz, $CDCl_3$) of **6**.

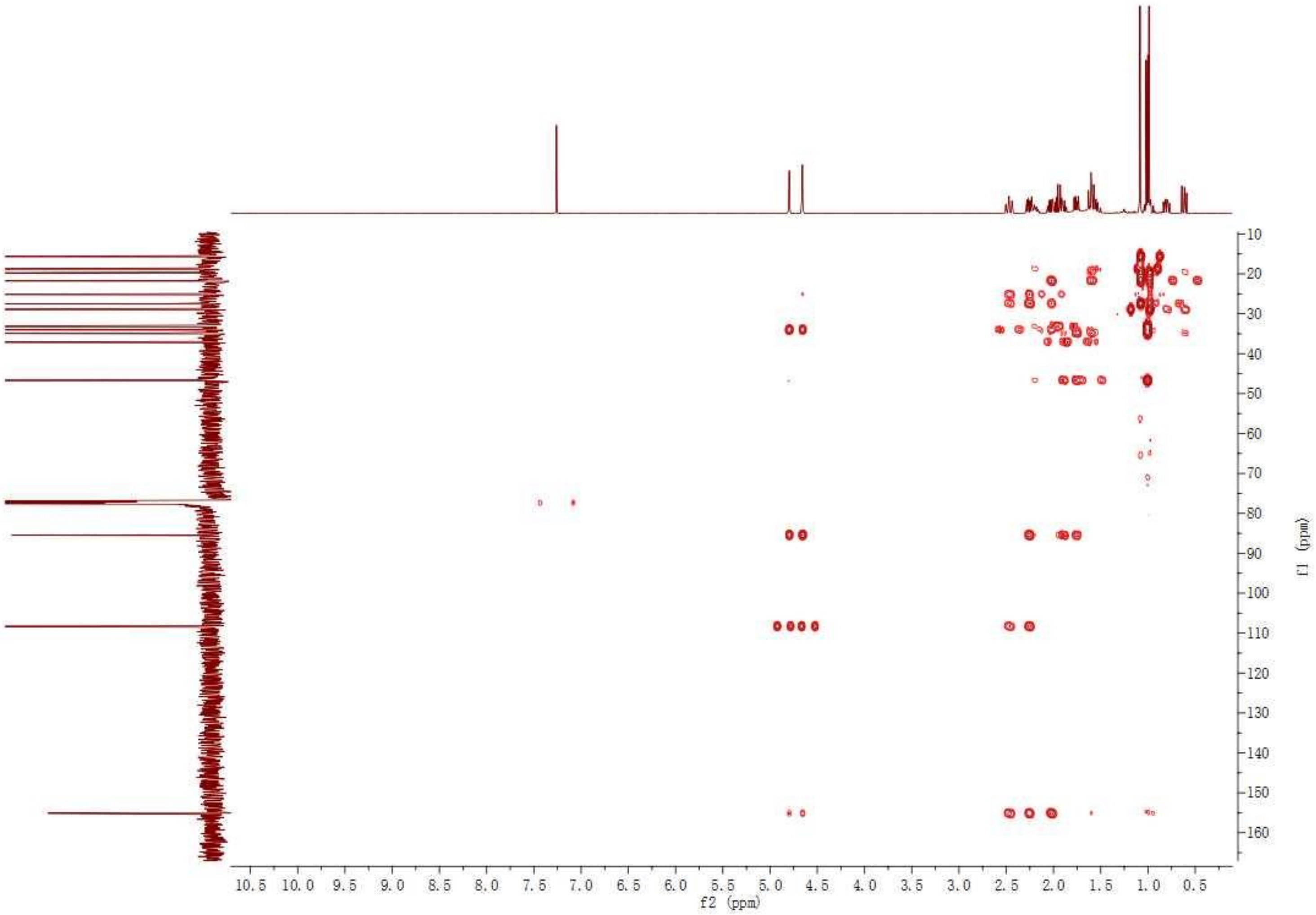

Figure S38: NOESY spectrum (500 MHz, $CDCl_3$) of **6**.

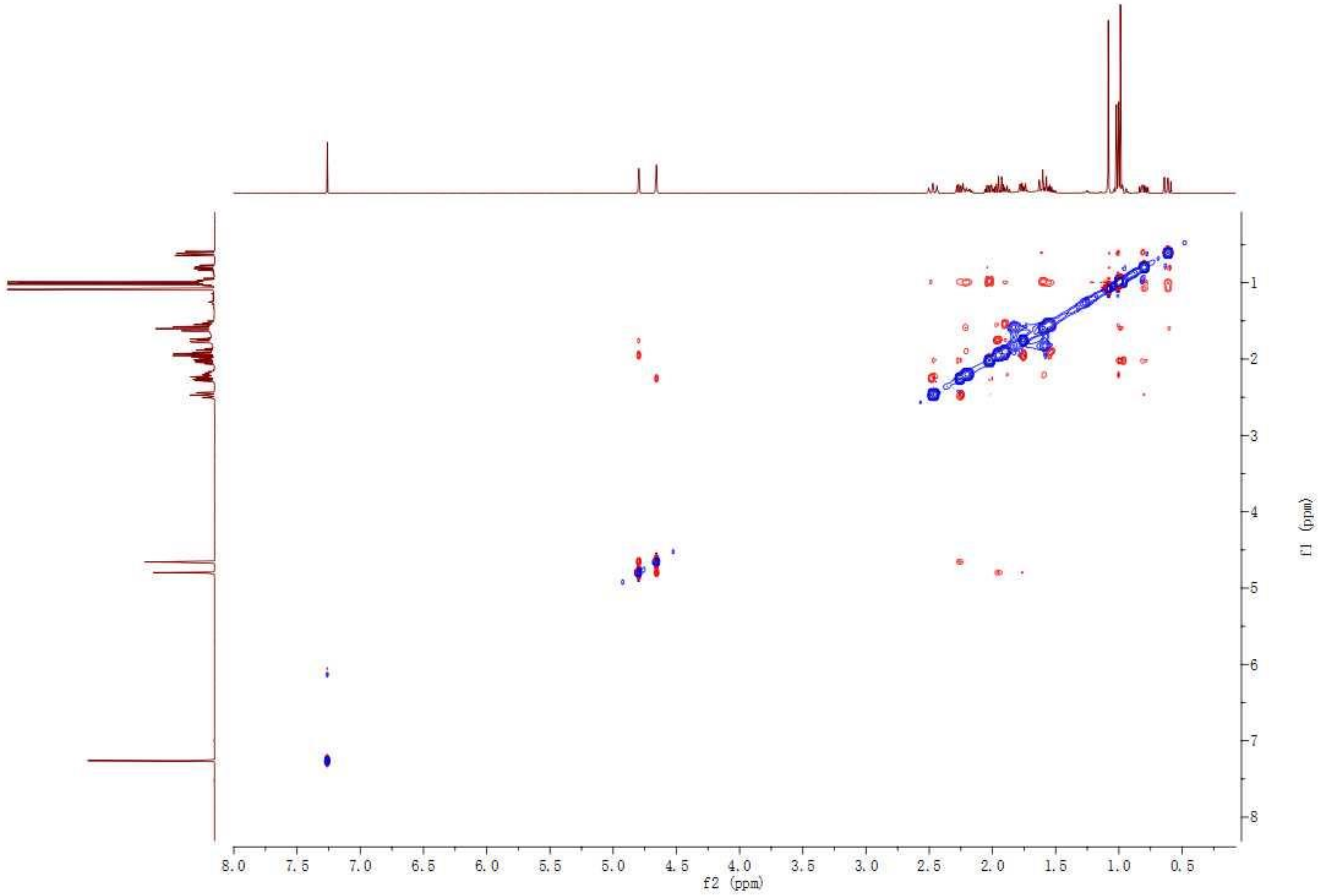

Figure S39: IR spectrum of **6**.

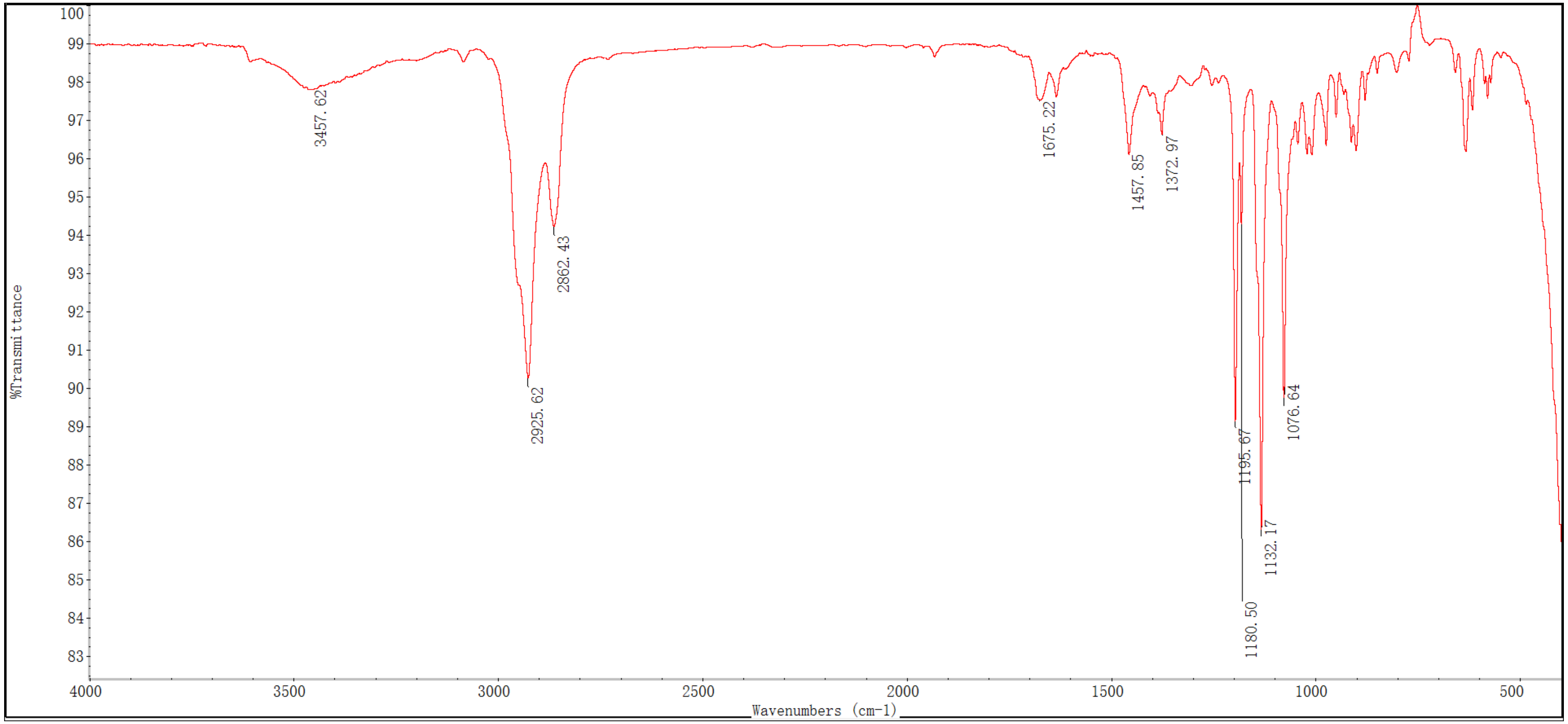

Figure S40: HR-EIMS spectrum of **6**.

LIST: h16377-c1 18-Sep-16 Elapse: 03:29.7 16
Samp: 21-F3b Start : 15:04:10 20
Comm: Finnigan/MAT95/70eV/R:10000
Mode: EI +VE +LMR BSCAN (EXP) UP HR NRM Study : S/N: PT200712-01-01
Oper: SIMM.CAS Client: S/N: PT001263 Inlet :
Limt: (0) . .
: (321) C17.H100.O
Peak: 1000.00 mmu R+D: -2.0 > 60.0
Data: CMASS : converted

		81092			(mmu)		
Mass		Intensity	%RA	%RIC	Delta	R+D	Composition
94.07697	*	114418	8.47	0.35	1.3	3.0	C7.H10
95.04998	*	168308	12.45	0.51	-0.3	3.5	C6.H7.O
95.08616	*	392362	29.03	1.20	-0.1	2.5	C7.H11
96.09313	*	165239	12.23	0.50	0.8	2.0	C7.H12
97.06499	*	94503	6.99	0.29	0.3	2.5	C6.H9.O
97.10086	*	98786	7.31	0.30	0.9	1.5	C7.H13
105.0674	*	786509	58.19	2.40			
106.0737	*	154461	11.43	0.47			
107.0463	*	84653	6.26	0.26			
107.0830	*	646823	47.86	1.97			
108.0891	*	131762	9.75	0.40			
109.0624	*	259600	19.21	0.79	3.0	3.5	C7.H9.O
109.0992	*	231620	17.14	0.71	2.6	2.5	C8.H13
115.0533	*	148393	10.98	0.45	1.5	6.5	C9.H7
117.0692	*	461027	34.11	1.40	1.3	5.5	C9.H9
118.0758	*	97430	7.21	0.30	2.5	5.0	C9.H10
119.0853	*	447680	33.12	1.36	0.8	4.5	C9.H11
120.0926	*	237830	17.60	0.72	1.3	4.0	C9.H12
121.0652	*	133832	9.90	0.41	0.2	4.5	C8.H9.O
121.1013	*	417915	30.92	1.27	0.5	3.5	C9.H13
122.0724	*	141684	10.48	0.43	0.8	4.0	C8.H10.O
122.1089	*	293219	21.70	0.89	0.7	3.0	C9.H14
123.0793	*	213847	15.82	0.65	1.7	3.5	C8.H11.O
123.1155	*	122269	9.05	0.37	1.8	2.5	C9.H15
125.0956	*	174232	12.89	0.53	1.1	2.5	C8.H13.O
128.0628	*	110492	8.18	0.34	-0.2	7.0	C10.H8
129.0699	*	141184	10.45	0.43	0.5	6.5	C10.H9
131.0864	*	642326	47.53	1.96	-0.3	5.5	C10.H11
132.0922	*	123483	9.14	0.38	1.7	5.0	C10.H12
133.1020	*	352676	26.09	1.07	-0.3	4.5	C10.H13
134.1082	*	147965	10.95	0.45	1.3	4.0	C10.H14
135.0805	*	132119	9.78	0.40	0.5	4.5	C9.H11.O
135.1166	*	214632	15.88	0.65	0.8	3.5	C10.H15
137.0962	*	134118	9.92	0.41	0.4	3.5	C9.H13.O
138.1038	*	135189	10.00	0.41	0.7	3.0	C9.H14.O
144.0939	*	88650	6.56	0.27	0.0	6.0	C11.H12
145.1017	*	654318	48.41	1.99	0.0	5.5	C11.H13
146.1076	*	205495	15.20	0.63	2.0	5.0	C11.H14
147.1169	*	270164	19.99	0.82	0.5	4.5	C11.H15
149.0965	*	252962	18.72	0.77	0.2	4.5	C10.H13.O
149.1327	*	110349	8.16	0.34	0.4	3.5	C11.H17
150.1034	*	206780	15.30	0.63	1.1	4.0	C10.H14.O
151.1124	*	343254	25.40	1.05	-0.1	3.5	C10.H15.O
159.1175	*	1351534	100.00	4.12	-0.2	5.5	C12.H15
160.1226	*	282655	20.91	0.86	2.6	5.0	C12.H16
161.1323	*	115488	8.54	0.35	0.7	4.5	C12.H17
162.1403	*	100142	7.41	0.31	0.5	4.0	C12.H18
163.1123	*	367665	27.20	1.12	0.0	4.5	C11.H15.O
164.1190	*	122198	9.04	0.37	1.1	4.0	C11.H16.O
173.1331	*	84582	6.26	0.26	0.0	5.5	C13.H17
177.1277	*	442540	32.74	1.35	0.2	4.5	C12.H17.O
178.1343	*	101927	7.54	0.31	1.5	4.0	C12.H18.O
187.1492	*	576802	42.68	1.76	-0.5	5.5	C14.H19
191.1438	*	132548	9.81	0.40	-0.2	4.5	C13.H19.O
202.1720	*	551748	40.82	1.68	0.2	5.0	C15.H22
205.1585	*	273661	20.25	0.83	0.7	4.5	C14.H21.O
220.1824	*	240756	17.81	0.73	0.3	4.0	C15.H24.O
